# Functional redundancy in tRNA dihydrouridylation

**DOI:** 10.1093/nar/gkae325

**Published:** 2024-04-29

**Authors:** Claudia Sudol, Lea-Marie Kilz, Virginie Marchand, Quentin Thuillier, Vincent Guérineau, Catherine Goyenvalle, Bruno Faivre, Sabrine Toubdji, Murielle Lombard, Olivier Jean-Jean, Valérie de Crécy-Lagard, Mark Helm, Yuri Motorin, Damien Brégeon, Djemel Hamdane

**Affiliations:** Sorbonne Université, CNRS, Institut de Biologie Paris Seine, Biology of Aging and Adaptation, Paris 75252, France; Collège De France, Sorbonne Université, CNRS, Laboratoire de Chimie des Processus Biologiques, 11 place Marcelin Berthelot, 75231 Paris Cedex 05, France; Institut für pharmazeutische und biomedizinische Wissenschaften (IPBW), Johannes Gutenberg-Universität, Mainz 55128, Germany; Université de Lorraine, CNRS, INSERM, UMS2008/US40 IBSLor, EpiRNA-Seq Core Facility, Nancy F-54000, France; Université de Lorraine, CNRS, UMR7365 IMoPA, Nancy F-54000, France; Université de Lorraine, CNRS, INSERM, UMS2008/US40 IBSLor, EpiRNA-Seq Core Facility, Nancy F-54000, France; Université de Lorraine, CNRS, UMR7365 IMoPA, Nancy F-54000, France; Université Paris-Saclay, CNRS, Institut de Chimie des Substances Naturelles, UPR 2301, 91198, Gif-sur-Yvette, France; Sorbonne Université, CNRS, Institut de Biologie Paris Seine, Biology of Aging and Adaptation, Paris 75252, France; Collège De France, Sorbonne Université, CNRS, Laboratoire de Chimie des Processus Biologiques, 11 place Marcelin Berthelot, 75231 Paris Cedex 05, France; Sorbonne Université, CNRS, Institut de Biologie Paris Seine, Biology of Aging and Adaptation, Paris 75252, France; Collège De France, Sorbonne Université, CNRS, Laboratoire de Chimie des Processus Biologiques, 11 place Marcelin Berthelot, 75231 Paris Cedex 05, France; Collège De France, Sorbonne Université, CNRS, Laboratoire de Chimie des Processus Biologiques, 11 place Marcelin Berthelot, 75231 Paris Cedex 05, France; Sorbonne Université, CNRS, Institut de Biologie Paris Seine, Biology of Aging and Adaptation, Paris 75252, France; Department of Microbiology and Cell Science, University of Florida, Gainesville, FL 32611, USA; University of Florida, Genetics Institute, Gainesville, FL 32610, USA; Institut für pharmazeutische und biomedizinische Wissenschaften (IPBW), Johannes Gutenberg-Universität, Mainz 55128, Germany; Université de Lorraine, CNRS, INSERM, UMS2008/US40 IBSLor, EpiRNA-Seq Core Facility, Nancy F-54000, France; Université de Lorraine, CNRS, UMR7365 IMoPA, Nancy F-54000, France; Sorbonne Université, CNRS, Institut de Biologie Paris Seine, Biology of Aging and Adaptation, Paris 75252, France; Collège De France, Sorbonne Université, CNRS, Laboratoire de Chimie des Processus Biologiques, 11 place Marcelin Berthelot, 75231 Paris Cedex 05, France

## Abstract

Dihydrouridine (D) is a common modified base found predominantly in transfer RNA (tRNA). Despite its prevalence, the mechanisms underlying dihydrouridine biosynthesis, particularly in prokaryotes, have remained elusive. Here, we conducted a comprehensive investigation into D biosynthesis in *Bacillus subtilis* through a combination of genetic, biochemical, and epitranscriptomic approaches. Our findings reveal that *B. subtilis* relies on two FMN-dependent Dus-like flavoprotein homologs, namely DusB1 and DusB2, to introduce all D residues into its tRNAs. Notably, DusB1 exhibits multisite enzyme activity, enabling D formation at positions 17, 20, 20a and 47, while DusB2 specifically catalyzes D biosynthesis at positions 20 and 20a, showcasing a functional redundancy among modification enzymes. Extensive tRNA-wide D-mapping demonstrates that this functional redundancy impacts the majority of tRNAs, with DusB2 displaying a higher dihydrouridylation efficiency compared to DusB1. Interestingly, we found that *Bs*DusB2 can function like a *Bs*DusB1 when overexpressed *in vivo* and under increasing enzyme concentration *in vitro*. Furthermore, we establish the importance of the D modification for *B. subtilis* growth at suboptimal temperatures. Our study expands the understanding of D modifications in prokaryotes, highlighting the significance of functional redundancy in this process and its impact on bacterial growth and adaptation.

## Graphical Abstract

**Graphical Abstract:**
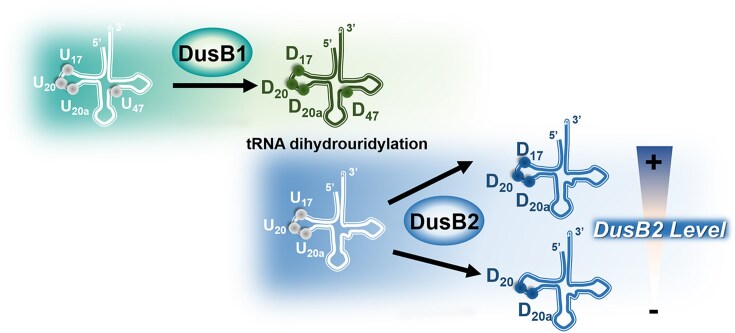

## Introduction

All RNA transcripts undergo a series of post-transcriptional processes tailored to optimize their functionality (1–3). These processes include the addition of various chemical groups appended to the base and/or ribose moieties at conserved positions within the RNA polymer, and catalyzed *de novo* by specific enzymes (4). Over 170 chemical modifications have been documented thus far, with ongoing advancements in transcriptome analysis, particularly through high-throughput sequencing technologies combined with chemical labeling and mass spectrometry, continually unveiling novel modifications (5). At the forefront of the most extensively modified RNA species lie tRNAs, small non-coding RNA molecules involved in decoding genetic information during translation (6,7). tRNAs undergo complex modifications predominantly clustered at positions 34 and 37 within the anticodon loop. These modifications are not only acknowledged for their indispensable role in ensuring the accuracy and efficiency of translation processes (8), but are also emerging as vital regulatory elements (9,10). Equally significant, the chemical modifications located outside the anticodon and scattered throughout the polymer stabilize the peculiar and essential L-shaped tRNA structure formed by the kissing dihydrouridine (D) and ribothymidine (rT = m^5^U at position 54) loops (11–13), both of which represent conserved modified bases.

Unlike all other modified bases, D is a non-aromatic base that cannot participate in stacking interactions or engage in base pairing via hydrogen bonding. Nevertheless, dihydrouridine fulfills a distinctive role by promoting the flexible C2'-endo conformation of the ribose (14). The exact function of D in RNA remains somewhat elusive, although several assumptions have been proposed. It is widely accepted that because the D base is not aromatic and thus disinclined to stacking interactions, it confers a certain degree of flexibility around its position, thereby allowing favorable tertiary interactions in the tRNA elbow region (14,15). This notion of flexibility finds support in studies showing that psychrophilic organisms, thriving in low-temperature environments, generally exhibit higher D content than thermophilic counterparts (16). In addition, higher D content may confer a growth advantage to cancer cells over healthy cells, perhaps by enhancing translational efficiency (17). However, the exact mechanisms underlying such effect remain unclear and require further exploration.

**Figure 1.**
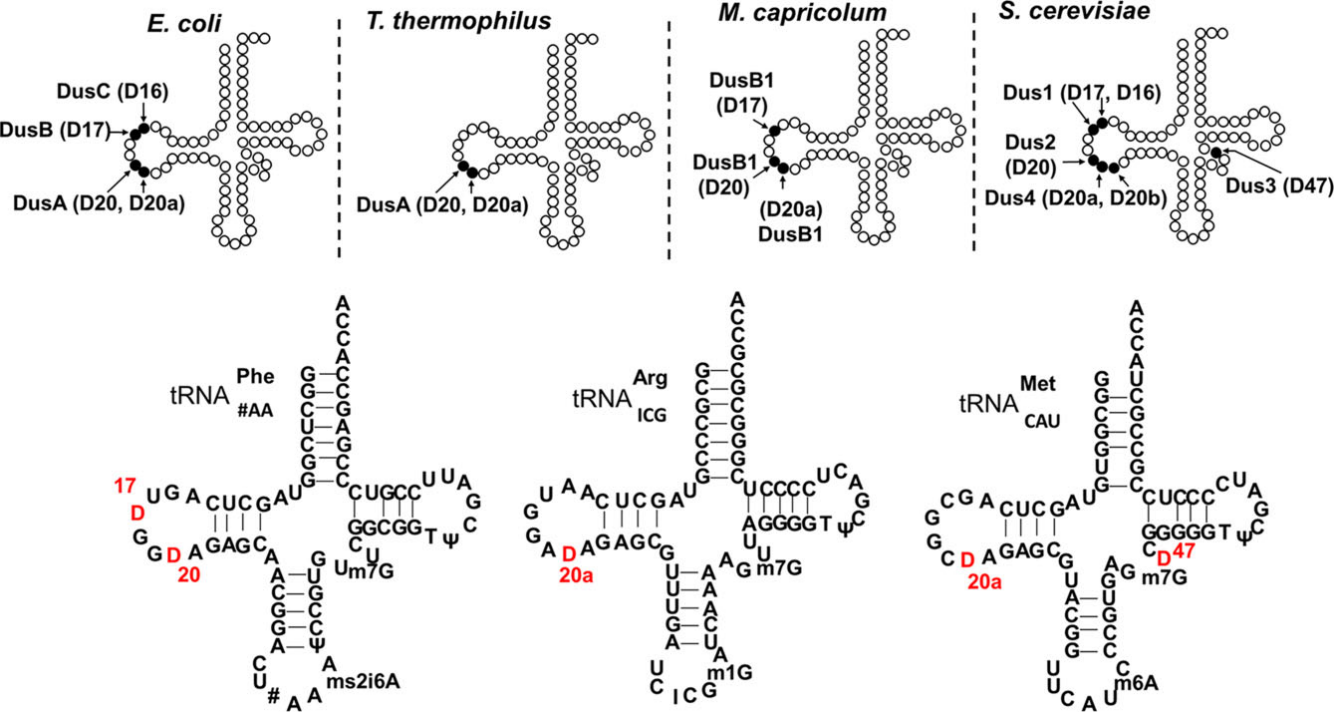
Location of D-sites in tRNA and the corresponding enzyme involved in site dihydrouridylation determined experimentally. Schematic representation of the secondary structure of tRNA, showing the location of D residues and the corresponding Dus enzyme responsible for their synthesis in *E. coli*, *T. thermophilus* and *M. capricolum* for eubacteria and *S. cerevisiae* for eukaryotes. In the lower panel is shown the sequence of *B. subtilis* tRNAs used to analyze the D-sites in the MALDI-MS experiments.

D is commonly present at multiple canonical sites in tRNAs (D16–D17–D20–D20a–D20b–D47), for both bacteria and eukaryotes (Figure 1), with its abundance depending on the organism and tRNA type (4,30). Its biosynthesis is achieved through the reduction of the C5=C6 uridine double bond, catalyzed by the dihydrouridine synthases (Dus), which belong to the COG0042 (Cluster of Orthologous Group) family of flavoenzymes (18–23). All hitherto investigated Dus enzymes use NADPH as a hydride source to reduce flavin mononucleotide (FMN) to FMNH^−^, which then donates its hydride to the electrophilic C6 atom of the uridine substrate (22,24). Initially regarded as a modified base commonly seen in tRNA, recent studies have reported D residues in mRNA and certain long non-coding RNAs in yeast and human cells (23,25–27). Dus enzymes responsible for introducing D into tRNA also participate in mRNA dihydrouridylation, highlighting their substrate promiscuity, a property shared with other RNA-modifying enzymes such as pseudouridine synthases, m^1^A and m^5^C methyltransferases (28,29). The site-specificities of the Dus enzymes have been established in various organisms including yeast (*Saccharomyces cerevisiae*, *Schizosaccharomyces pombe*), humans, *Escherichia coli*, *Thermus thermophilus* and recently in *Mycoplasma capricolum* (18,26,30–33) (Figure 1). These enzymes have been categorized into eight subfamilies, including three bacterial Dus (DusA, DusB, DusC), four eukaryotic Dus (Dus1, Dus2, Dus3, Dus4), and an archaeal Dus (34). The distribution of Dus enzymes is less uniform in prokaryotes and varies between organisms. For instance, Gammaproteobacteria such as *E. coli* possess the three bacterial Dus enzymes, namely DusA, B and C, involved in D20-D20a, D17 and D16, modifications (Figure 1), respectively (31). In contrast, *T. thermophilus* has only one Dus enzyme of the DusA type, which synthesizes the D20–D20a modifications (32) (Figure 1).

DusB emerged as the first Dus from the ancestral Dus, giving rise subsequently to DusA and DusC through duplication events (34). Our recent phylogenetic analysis revealed that Gram-positive bacteria exclusively carry DusB homologs, categorized into three subgroups: DusB1, DusB2 and DusB3 (33). While most of the examined genomes carry either a *dusB1* or *dusB2* gene, approximately 40% of these organisms contain both *dusB1* and *dusB2* genes, with *dusB3* being restricted to a subset of Clostridia. *Bacillus* species generally retained both *dusB1* and *dusB2* (BSU00810 and BSU08030 annotated as *dusB* and *dusC*, respectively), while Mollicutes conserved only *dusB1* and *Staphylococcus* species kept *dusB2*. Both DusB subgroups likely originated from an ancestral DusB duplication event, which probably occurred in the common ancestor of the Firmicutes. In addition, the limited distribution of DusB3 suggests more recent origin. Biochemical characterization of DusB1 from *M. capricolum* (MCAP_0837) revealed its multisite specificity, catalyzing dihydrouridylation at U17, U20 and U20a positions, (Figure 1), consistent with sequenced tRNAs from this Mollicute species (33). The multi-site specificity feature of Gram-positive Dus, likely shared by both DusB1 and DusB2, is also supported by the tRNA modification profiles of three other bacteria: *Lactococcus lactis*, *Streptomyces griseus* and *S. aureus*, all displaying D17, D20 and D20a modifications.

All cases studied show that a given D residue is specifically synthesized by a single Dus. However, while several Dus have been reported to synthesize D at different positions, the redundancy of synthesis in terms of overlapping specificities has not yet been documented. In this investigation, we explore the contribution of Dus homologs, specifically DusB1 and DusB2, in D biosynthesis. Using *B. subtilis* as our model organism, we reveal a significant level of functional redundancy in D biosynthetic pathways catalyzed by both DusB homologs.

## Materials and methods

### Deletion of *dusB1* and *dusB2* of *B. subtilis* and complementation

The *B. subtilis* strains used in this study were derived from strain W168, obtained from Chastanet's lab (INRAE, Jouy en Josas, France), and listed in Supplementary Table S1. All the primers used for mutant strain and plasmid constructions in this study are listed in Supplementary Table S2. Mutant strains were obtained from Bacillus Genetic Stock Center. Double mutant Δ*dusB1::kan*, Δ*dusB2::erm* strain was generated by transforming single Δ*dusB1::kan* with the PCR product amplified from the single Δ*dusB2::erm* genome using BSU08030-5pL/BSU08030-3pR (35). *B. subtilis* strains expressing *SadusB2* (SACOL0067) under the control of *BsdusB1* promoter (Δ*dusB1::SadusB2-kan*, Δ*dusB2::erm*) was obtained by transforming Δ*dusB2::erm* strain with a PCR fragment containing (i) the 5′ *BsdusB1* genomic sequence, (ii) *SadusB2* CDS, (iii) a kanamycin resistance cassette and (iv) the 3′ *BsdusB1* genomic sequence. The same strategy was used to express *McdusB1* (MCAP_0837). All Bacillus transformations were performed following the protocol described by Koo *et al.* (35). Strain selections were done on LB-agar containing kanamycin (40 μg ml^−1^) and/or erythromycin (5 μg ml^−1^). All strains were verified by PCR and sequencing. *E. coli* strains and growth conditions are detailed in previous studies (31,33).

### Cloning *dus*B1 and *dus*B2 from *B. subtilis*, *dus*B1 from *M. capricolum* and *dus*B2 from *S. aureus*

Plasmids containing *dusB1* and *dusB2* genes of *B. subtilis* (pEX-*BsdusB*1 and pEX-*BsdusB2*) and *dusB2* of *S. aureus* (pEX- *SadusB2*) were obtained from Eurofins. We used these plasmids to amplify by PCR *dusB* gene sequences using the primer pairs listed in Supplementary Table S2. The *dusB1* and *dusB2* genes of *B. subtilis* were cloned as follow into pET15b with a sequence encoding for a 6-histidine tag and a thrombin protease site placed at the 5′ end of the genes. After amplification, PCR fragments purified with QIAquick PCR purification kit (Qiagen) were cloned into PCR-linearized pET15b plasmid using the SLIC cloning method (36). Similarly, *dusB1* and *dusB2* genes of *B. subtilis* were cloned in pDG148 for overexpression in *B. subtilis* strains (37). In the case of *dusB2* from *S. aureus* (*SadusB2*), the gene was cloned into the pET28a plasmid containing a sequence encoding for a 6-histidine tag placed at 5′ end of *SadusB2* gene using the same strategy as described above. After cloning, *dusB* gene integrity was verified by DNA sequencing (Eurofins).

### RNA extraction and tRNA purification

Bulk tRNA was extracted from *B. subtilis* W168, and its derivative Δ*dusB1::kan* and Δ*dusB2::erm* or double mutant Δ*dusB1::kan*, Δ*dusB2::erm*. Purification of specific tRNA has been previously described (31). Here, ${\mathrm{tRNA}}_{{\mathrm{ICG}}}^{{\mathrm{Arg}}}$, ${\mathrm{tRNA}}_{{\mathrm{GAA}}}^{{\mathrm{Phe}}}$ and ${\mathrm{tRNA}}_{{\mathrm{CAU}}}^{{\mathrm{Met}}}$, from *B. subtilis* strains was performed with 5′ biotinylated complementary oligonucleotide (5′-biot-TGGCGCGCCCGAGGGGAGTCGAACCCCTAA-3′, 5′-biot-TGGTGGCTCGGGACGGAATCGAACCGCCGA-3′ and 5′-biot-TGGTAGCGGCGGAGGGGATCGAACCCCCG-3′ respectively) while ${\mathrm{tRNA}}_{{\mathrm{ICG}}}^{{\mathrm{Arg2}}}$, ${\mathrm{tRNA}}_{{\mathrm{GAU}}}^{{\mathrm{Ile1}}}$ and ${\mathrm{tRNA}}_{{\mathrm{CAG}}}^{{\mathrm{Leu1}}}$, from *E. coli* were purified as described previously (31). For AlkAniline-Seq and LC-MS experiments, total RNA was isolated using hot phenol or Trizol according to manufacturer's instructions.

### Activity assay and dihydrouridine quantification

*In vitro* activity was assayed for 1 h at 37°C in 50 mM HEPES pH 7.5, 150 mM NaCl, 5 mM DTT, 10 mM MgCl_2_, 100 μM FMN and 15% glycerol under air. Bulk tRNAs (25 μM) issued from the Δ*dusB1::kan*, Δ*dusB2::erm* strain were incubated with various concentration of protein ranging from 0.05 to 50 μM in a total volume of 100 μl and reaction was started upon addition of NADPH at a final concentration of 2 mM. Quenching was performed by adding 100 μl of acidic phenol (Sigma-Aldrich) followed by centrifugation at 16 000×*g* for 10 min. tRNAs in the aqueous phase were ethanol precipitated and further purified using a MicroSpin G-25 column (GE-healthcare). Dihydrouridine quantification was carried out by LC–MS spectrometry analysis.

### Liquid chromatography–tandem mass spectrometry (LC–MS)

1 μg of tRNA per sample was digested to nucleoside level using 0.6 units (U) nuclease P1 from *P. citrinum* (Sigma-Aldrich), 0.2 U snake venom phosphodiesterase from *C. adamanteus* (Worthington), 2 U FastAP (Thermo Scientific), 10 U benzonase (Sigma-Aldrich) and 200 ng Pentostatin (Sigma-Aldrich) in 25 mM ammonium acetate buffer at pH 7.5 (Sigma-Aldrich) overnight at 37°C. LC–MS/MS analysis was performed using an Agilent 1260 series LC with a Synergi Fusion RP18 column (4 μM particle size, 80 Å pore size, 250 × 2.0 mm; Phenomenex) and an Agilent 6460A Triple Quadrupole mass spectrometer equipped with an electrospray ion source (ESI). 5 mM ammonium acetate buffer at pH 5.3 was used as solvent A and LC–MS grade acetonitrile (Honeywell) served as solvent B. The elution started with 100% solvent A with a flow rate of 0.35 mL/min, followed by a linear gradient to 8% solvent B at 10 min, raising to 40% solvent B after 20 min and subsequent three-minute restoration of the initial conditions. 100% solvent A was held for further 10 min before starting the next elution. During elution a diode array detector (DAD) recorded the UV signal at 254 nm to monitor the main nucleosides and the ESI parameters were set as follows: gas temperature 350°C, gas flow 8 l min^−1^, nebulizer pressure 50 psi, sheath gas temperature 350°C, sheath gas flow 12 l min^−1^ and capillary voltage 3000 V. The mass spectrometer was run in the dynamic multiple reaction monitoring (dMRM) mode using Agilent MassHunter software. The quantitative analysis was performed as described in Kellner *et al.* (38) using internal calibration. For internal calibration 300 ng of digested sample were spiked with 50 ng of 13C stable isotope-labelled nucleosides from *E. coli* and subjected to analysis.

### MALDI-TOF spectrometry analysis

For mass spectrometry analysis, about 50 μg of tRNAs were digested with either 10 μg of RNAse A (Euromedex) or RNAseT1 (Sigma-Aldrich), which generates 3′-phosphate nucleosides, in a final volume of 10 μl at 37°C for 4 h. One microliter of digest was mixed with 9 μl HPA (40 mg/ml in water: acetonitrile 50:50) and 1 μl of the mixture was spotted on the MALDI plate and air-dried (‘dried droplet’ method) as previously described (31). MALDI-TOF MS analyses were performed directly on the digestion products using an UltrafleXtreme spectrometer (Bruker Daltonique, France). Acquisitions were performed in positive ion mode. An identical strategy was applied for RNase T1 digests (cleavage after G generating 3′-phosphate nucleosides).

### Bioinformatic analyses

The FASTA sequences of 203 proteins annotated in BV-BRC (39) as tRNA-dihydrouridine (20/20a) synthase (EC 1.3.1.91) (or DusA), tRNA-dihydrouridine (16) synthase (or DusC), tRNA-dihydrouridine synthase DusB (or DusB/DusB1) and tRNA-dihydrouridine synthase 2 (or DusB2) where extracted from 120 reference bacterial genomes using the BV-BRC filtering tools. A phylogenetic tree was generated using the MAFFT version 7 (40) web-based pipeline (https://mafft.cbrc.jp/alignment/server/) using the default parameters with boostrap sampling size of 100. The final newick tree is given as Supplemental data 2. The newick tree was then visualized and annotated in iTol (41).

## Results

### Contribution of *Bs*DusB1 and *Bs*DusB2 to tRNA dihydrouridylation in *B. subtilis*

*B. subtilis*, complete modification profiles have been established for 24 tRNA sequences over a total of 35 different iso-acceptors, allowing us to compile a more or less accurate distribution of the D sites present in this organism (4). The predominant positions where D is found include the canonical positions 17, 20 and 20a, along with position 47 for a single tRNA, ${\mathrm{tRNA}}_{{\mathrm{CAU}}}^{{\mathrm{Met}}}$. A quick survey shows that residues D20 and D20a are the most frequent D residues, followed by D17 and D47 (Supplementary Table S3). Notably, D20 stands out as the most prevalent across all tRNA sequences from all organisms (4). While the abundance of each specific tRNA still needs to be determined, it is reasonable to assume that D20 and D20a account for most of the D content in *B. subtilis* tRNAs.

**Figure 2.**
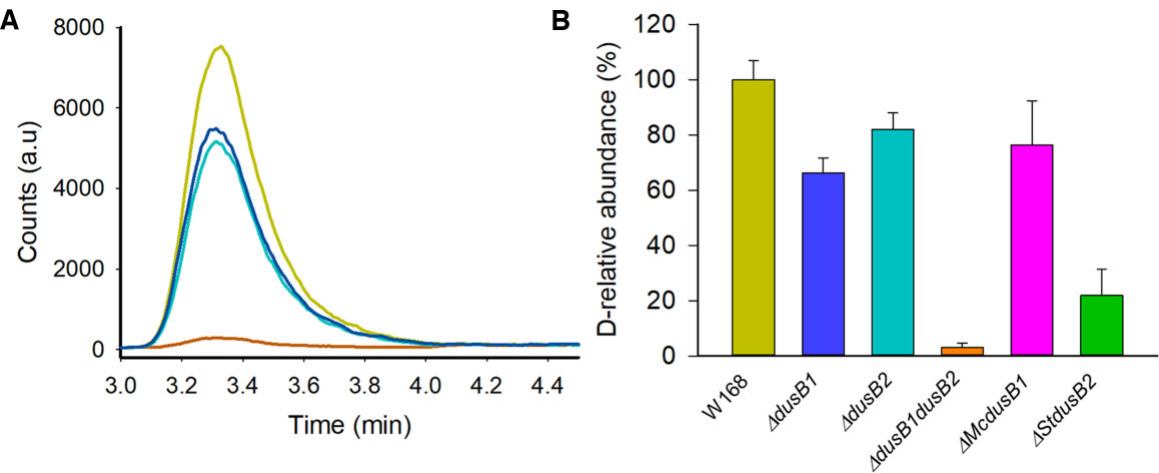
Quantification of D-level in tRNA from *B. subtilis*. (**A**) Extracted ion chromatograms of dihydrouridine in tRNAs isolated from *B. subtilis* WT strain (W168 in light green), Δ*dusB1::kan*, Δ*dusB2::erm* double deletion strain (orange) and Δ*dusB1::kan* (blue) and Δ*dusB2::erm* single mutant strains (cyan). The signals were normalized to the respective UV signal of Adenosine. (**B**) D levels determined in bulk tRNAs of *B. subtilis* WT strains (W168 in light green), Δ*dusB1* (blue) and Δ*dusB2* (cyan) single mutants, Δ*dusB1*Δ*dusB2* (orange) or double deletion complemented with either DusB1 of *M. capricolum* (magenta) or DusB2 from *S. aureus* (green). The strains were grown in LB media at 37°C. Results are shown as average of three biological replicates in relation to the *wild type* strain W168.

The D content was determined using liquid chromatography–mass spectrometry (LC–MS) in tRNAs extracted from wild type *B. subtilis* W168 strain or from the isogenic single mutants (Δ*dusB1::kan* and Δ*dusB2::erm*) or double mutant (Figure 2A). The double deletion led to a complete depletion of D content in bulk *B. subtilis* tRNA, indicating that one or both DusB enzymes cover all D biosynthesis in tRNA. However, intriguingly, in the Δ*dusB1::kan* strain, D content decreased by 34%, while in the Δ*dusB2::erm* strain, it only decreased by 18%. In other words, in the Δ*dusB1::kan* strain, *Bs*DusB2 was responsible for 66% of the D content, whereas *Bs*DusB1 synthesized 82% of the D content in the Δ*dusB2::erm* strain (Figure 2B). These seemingly contradictory results may in fact be explained by an overlapping specificity shared by the two DusB enzymes. Complementation assays in the *B. subtilis* Δ*dusB1*::kan,Δ*dusB2*::erm strain showed that the expression of *McdusB1* and *SadusB2* from the *dusB1* promoter restored 76% and 22% of the D content of wild type tRNAs, respectively (Figure 2B). This indicates that both genes encode for Dus enzymes, and that the *M. capricolum* enzyme is more active than the *S. aureus* enzyme in the *B. subtilis* heterologous system.

### Functional redundancy of the DusB enzymes in *B. subtilis* determined by MALDI-MS

**Figure 3.**
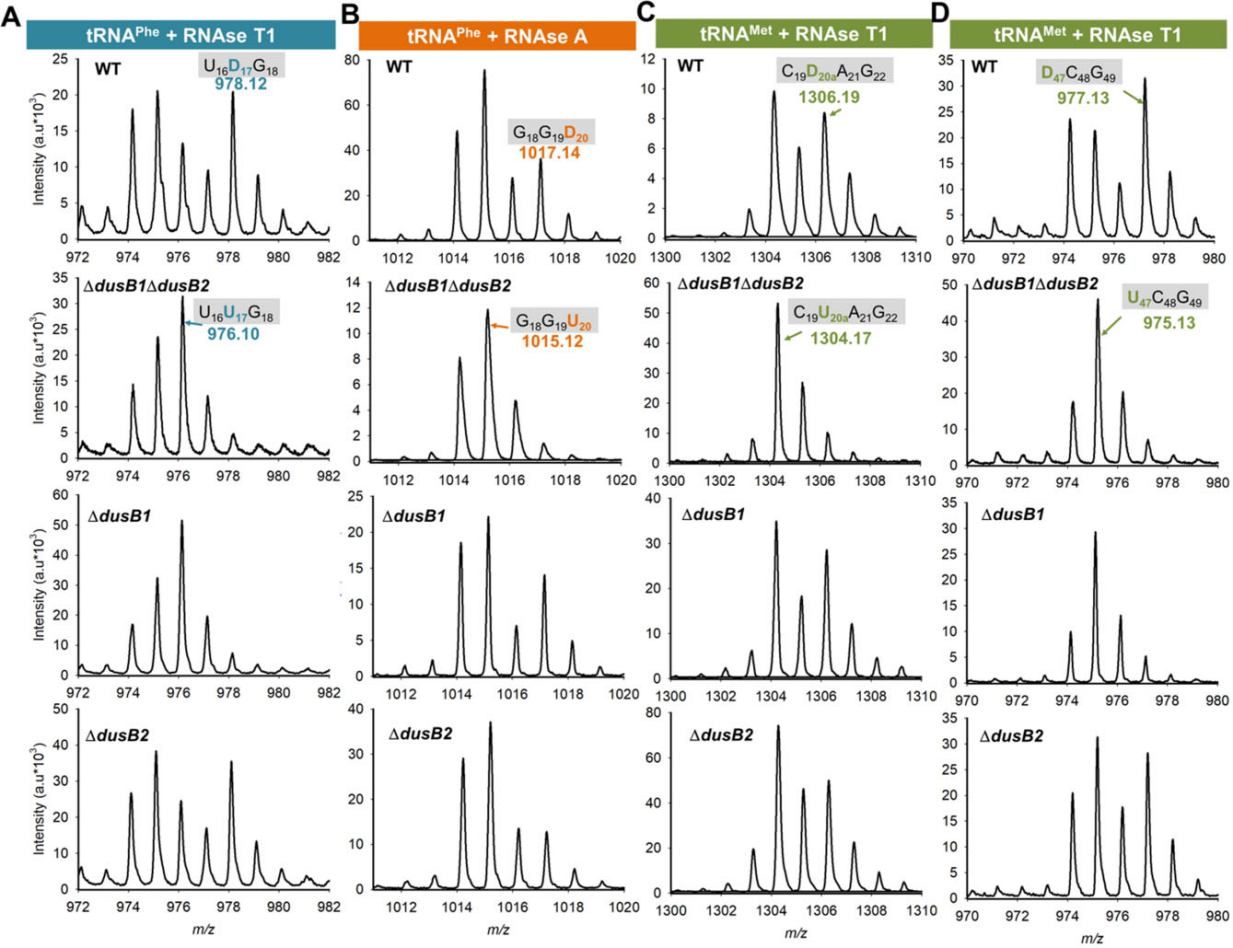
MALDI-TOF analysis of position 17, 20, 20a and 47 in tRNAs from *B. subtilis WT* and Dus deletion mutants. (**A**) D17-containing MS relative isotope patterns of derived oligonucleotides after RNAse T1 treatment of ${\mathrm{tRNA}}_{{\mathrm{\# AA}}}^{{\mathrm{Phe}}}$ isolated from wild type, Δ*dusB1*Δ*dusB2*, Δ*dusB1 and* Δ*dusB2*, respectively. (**B**) D20-containing MS relative isotope patterns of derived oligonucleotides after RNAse A treatment of ${\mathrm{tRNA}}_{{\mathrm{\# AA}}}^{{\mathrm{Phe}}}$ isolated from wild type, Δ*dusB1*, Δ*dusB2 and* Δ*dusB1*Δ*dusB2*, respectively. (**C**) D20a-containing MS relative isotope patterns of derived oligonucleotides after RNAse T1 treatment of ${\mathrm{tRNA}}_{{\mathrm{CAU}}}^{{\mathrm{Met}}}$ isolated from wild type, Δ*dusB1*, Δ*dusB2 and* Δ*dusB1*Δ*dusB2*, respectively. (**D**) D47-containing MS relative isotope patterns of derived oligonucleotides after RNAse T1 treatment of ${\mathrm{tRNA}}_{{\mathrm{CAU}}}^{{\mathrm{Met}}}$ isolated from wild type, Δ*dusB1*, Δ*dusB2 and* Δ*dusB1*Δ*dusB2*, respectively. Further details of the tRNA-derived oligonucleotide fragments and their sizes (*m/z*) used for the identification of DusB specificities are shown in supplementary figures.

The *in vivo* specificity of *Bs*DusB1 and *Bs*DusB2 dihydrouridylation sites was determined by comparing the D content in the tRNAs of four *B. subtilis* strains, including the W168 (wild type) and the single or double deletion strains. The approach involved a three-step workflow: (i) purification of specific tRNA types from various *B. subtilis* cells, (ii) fragmentation of the tRNA using RNAseA or RNAseT1 and (iii) analysis of the resulting fragments by MALDI-TOF. Deletion of *dusB* genes was expected to generate fragments containing U residues at the positions targeted by the corresponding enzymes, resulting in a −2Da shift relative to fragments in tRNAs extracted from wild type cells. We selected three tRNAs to cover all D sites, namely, ${\mathrm{tRNA}}_{{\mathrm{GAA}}}^{{\mathrm{Phe}}}$ for D17 and D20, ${\mathrm{tRNA}}_{{\mathrm{ICG}}}^{{\mathrm{Arg}}}$ for D20a and ${\mathrm{tRNA}}_{{\mathrm{CAU}}}^{{\mathrm{Met}}}$ for D20a and D47 (Figure 1). The mass profiles of these tRNAs are depicted in Figure 3A–D and Supplementary Figure S1. Analysis of tRNAs from the W168 strain confirmed the presence of all distinct D-containing fragments at the expected positions, validating the approach. Analysis of the D17 modification was made possible by monitoring the *m/z* 978 fragment corresponding to the UD_17_G trinucleotide generated by digestion of ${\mathrm{tRNA}}_{{\mathrm{GAA}}}^{{\mathrm{Phe}}}$ by RNAseT1 (Figure 3A). This fragment (its corresponding intensity showing background level) was absent in the double mutant strain while the intensity of the *m/z* 976 peak increased. Similar results were observed for ${\mathrm{tRNA}}_{{\mathrm{GAA}}}^{{\mathrm{Phe}}}$ from the Δ*dusB1::kan* strain. In contrast, the UD_17_G fragment was detected in the Δ*dusB2::erm* strain with intensity comparable to that of the W168 control. Therefore, these results suggested that *Bs*DusB1 was responsible for D17 biosynthesis. D20 was probed with two distinct fragments of D-containing ${\mathrm{tRNA}}_{{\mathrm{GAA}}}^{{\mathrm{Phe}}}$ from two different digestions. The first digestion, performed with RNAseA, yielded the GGD_20_ trinucleotide (*m*/*z* 1017) (Figure 3B). The second digestion, performed with RNAseT1, generated the trinucleotide D_20_AG (*m/z* 1001). In both scenarios, these two fragments did not disappear in ${\mathrm{tRNA}}_{{\mathrm{GAA}}}^{{\mathrm{Phe}}}$ from the two *dusB* single deletion strains, although a more consequent decrease in intensity was observed in the case of Δ*dusB2::erm*. In contrast, in the case of the double mutant, the peak was no longer detectable. We concluded that D20 was inserted into ${\mathrm{tRNA}}_{{\mathrm{GAA}}}^{{\mathrm{Phe}}}$ using both *Bs*DusB1 and *Bs*DusB2, with a dihydrouridylation efficiency that appeared to be higher for *Bs*DusB2. D20a was detected in two different tRNAs: ${\mathrm{tRNA}}_{{\mathrm{ICG}}}^{{\mathrm{Arg}}}$ via the GGAD_20a_ (*m/z* 1346) fragments obtained by RNAseA treatment and AD_20a_AG generated by RNAseT1 (*m/z* 1346) (Supplementary Figure S1), and ${\mathrm{tRNA}}_{{\mathrm{CAU}}}^{{\mathrm{Met}}}$ via the CD_20a_AG fragment (*m/z* 1306) obtained by RNAseT1 (Figure 3C). In the case of D20a in ${\mathrm{tRNA}}_{{\mathrm{CAU}}}^{{\mathrm{Met}}}$, both DusBs participated in its synthesis as neither mutant caused a substantial decrease in the intensity of the *m/z* 1306 peak, and their profiles were quite similar to that of the wild type. However, for D20a in ${\mathrm{tRNA}}_{{\mathrm{ICG}}}^{{\mathrm{Arg}}}$, only the deletion of *BsdusB2* or the double mutant led to a significantly decreased peak at *m/z* 1346, accompanied by an increase in the peak at *m/z* 1344 corresponding to the non-dihydrouridylated fragment (Supplementary Figure S1). These results suggest that the involvement of the two *Bs*DusB paralogs in D20a biosynthesis may depend on the tRNA substrate. Lastly, D47 was assayed by following the D_47_CG fragment (*m/z* 977), derived from treatment of ${\mathrm{tRNA}}_{{\mathrm{CAU}}}^{{\mathrm{Met}}}$ with RNAseT1 (Figure 3D). This analysis was carried out following the same analytical grid as before. The ${\mathrm{tRNA}}_{{\mathrm{CAU}}}^{{\mathrm{Met}}}$ from wild type and Δ*dusB2::erm B. subtilis* strains retained the prominent peak at *m/z* 977. In contrast, in the case of the Δ*dusB1::kan* or the double mutant strains, the intensity of this peak drastically decreased concomitantly with the increase in the *m/z* 975 peak, suggesting that *Bs*DusB1 was also responsible for D47 biosynthesis.

### Dihydrouridylation redundancy targets several tRNAs as investigated by deep-sequencing based AlkAnilineSeq method

An analysis of *B. subtilis Bs*DusB *in vivo* specificities was performed using the AlkAnilineSeq method (see supplementary methods for details) (42). This method exploits the D-ring's instability under alkaline conditions (20), leading to its cleavage and the formation of β-ureidopropionic acid. This instability results in aniline-driven RNA cleavage, generating a 5′-phosphate group (5′-P) on the neighboring N + 1 residue, which serves as an input for highly selective ligation of sequencing adapters. Alongside D-residue detection, AlkAnilineSeq also allows parallel detection of 7-methylguanosine (m^7^G), 3-methylcytidine (m^3^C) and 5-hydroxycytidine (ho^5^C), which share some degree of fragility in their base rings and/or N-glycosidic bonds, present in these modified residues. Mapping was achieved for all D containing tRNAs from the four *B. subtilis* strains, including the W168 strain, as well as the single and double *dus* deletion strains. It is important to emphasize that none of the D residues detected by this method was present at stoichiometric levels, suggesting partial dihydrouridylation of the target uridines. Importantly, the results obtained by AlkAnilineSeq were consistent with the MALDI-MS mapping experiments. For example, the disappearance of D17 in ${\mathrm{tRNA}}_{{\mathrm{GGC}}}^{{\mathrm{Ala}}}$ and ${\mathrm{tRNA}}_{{\mathrm{UGC}}}^{{\mathrm{Ala}}}$ of *B. subtilis* W168 was observed only in Δ*dusB1::kan* and double deletion strains, suggesting that *Bs*DusB1 was involved in the reduction of U17 in these tRNAs. In ${\mathrm{tRNA}}_{{\mathrm{ACG}}}^{{\mathrm{Arg}}}$, the loss of both D17 and D20a was seen in the double deletion strain, whereas in the Δ*dusB1::kan* strain, only the loss of D17 was observed (Supplementary Figure S2). In contrast, in the Δ*dusB2::erm* strain, the signal attributed to D20a declined when compared to the signal observed in Δ*dusB1::kan*, while D17 remained unchanged (Supplementary Figure S2). This is consistent with the fact that *Bs*DusB1 was responsible for the formation of both D17 and D20a, whereas *Bs*DusB2 formed only D20a in this tRNA (Supplementary Figure S2). Moreover, *Bs*DusB1 was implicated in the biosynthesis of all three D17/D20/D20a residues in ${\mathrm{tRNA}}_{{\mathrm{GUC}}}^{{\mathrm{Asp}}}$, whereas *Bs*DusB2 participated only in the latter two positions. In the case of ${\mathrm{tRNA}}_{{\mathrm{UUC}}}^{{\mathrm{Glu}}}$, *Bs*DusB1 was only capable of forming D20, while *Bs*DusB2 could form both D20 and D20a. These findings suggested that the two enzymatic dihydrouridylation activities did overlap.

**Figure 4.**
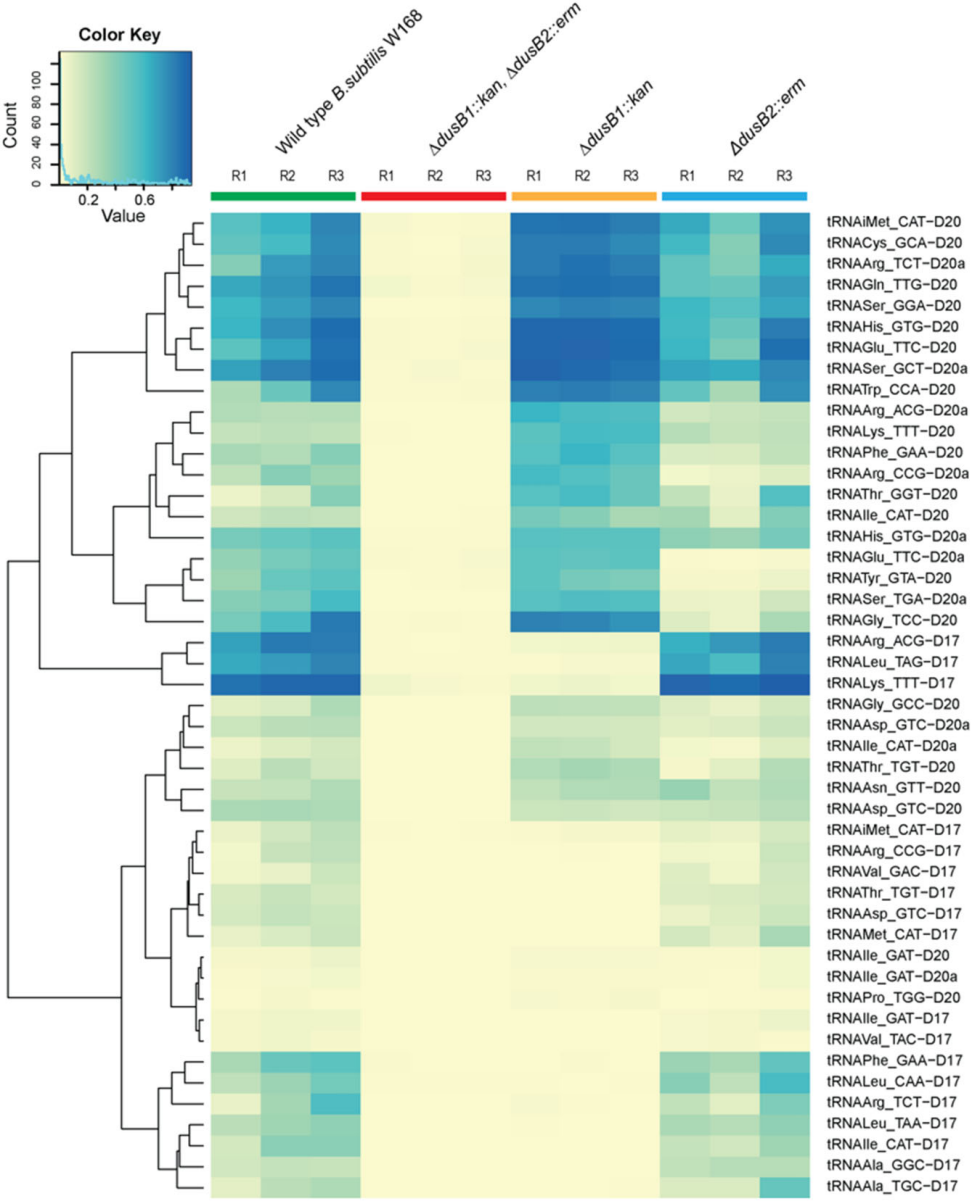
Heatmaps for the assessment of dihydrouridylation changes in individual modified sites in tRNAs from *B. subtilis* and its DusB mutants. The heatmap displays one specific D-modification's stoichiometry across the different samples (in X-axis) and the different D-sites retained for analysis (in Y-axis). The stoichiometry is blue-coded and relies on through stop ratio of the AlkAnilineSeq detection method, which detects m^7^G, m^3^C and D. R1, R2 and R3 represent the results for the three different replicas.

To gain a comprehensive view of both *Bs*Dus enzymes' activity, we generated an activity profile heatmap, as presented in Figure 4. The heatmap clearly demonstrates that only the double mutant lacked all D residues in tRNAs, consistent with both LC–MS and MALDI-TOF data. This supports the earlier observation that both Dus enzymes are essential for dihydrouridylation across the full range of tRNA substrates. Moreover, it is evident from the heatmap that only the *Bs*DusB1 enzyme was involved in the formation of D17, whereas both enzymes contributed to the formation of D20 and D20a. Further analysis of the D signal intensities revealed that while most of the D20 and D20a residues were synthetized by both *Bs*DusB1 and *Bs*DusB2, a few dihydrouridylation events preferentially used *Bs*DusB2 (such as for D20 in ${\mathrm{tRNA}}_{{\mathrm{UCC}}}^{{\mathrm{Gly}}}$ and ${\mathrm{tRNA}}_{{\mathrm{GUA}}}^{{\mathrm{Tyr}}}$ and for D20a in ${\mathrm{tRNA}}_{{\mathrm{CAU}}}^{{\mathrm{Ile}}},\ {\mathrm{tRNA}}_{{\mathrm{UGA}}}^{{\mathrm{Ser}}},\ {\mathrm{tRNA}}_{{\mathrm{CCG}}}^{{\mathrm{Arg}}}$). We did not detect D-signal for three tRNA, namely ${\mathrm{tRNA}}_{{\mathrm{GAU}}}^{{\mathrm{Ile}}}$, ${\mathrm{tRNA}}_{{\mathrm{UGG}}}^{{\mathrm{Pro}}}$ and ${\mathrm{tRNA}}_{{\mathrm{UAC}}}^{{\mathrm{Val}}}.$ Also the AlkAnilineSeq method did not detect the presence of D47, unlike the experiments performed by MALDI-MS on ${\mathrm{tRNA}}_{{\mathrm{CAU}}}^{{\mathrm{Met}}}$. This discrepancy could be explained by interference caused by m^7^G46, which produces a strong AlkAnilineSeq signal.

### *Bs*DusB1 and *Bs*DusB2 are flavoproteins characterized by a distinct polarity of their active site

**Figure 5.**
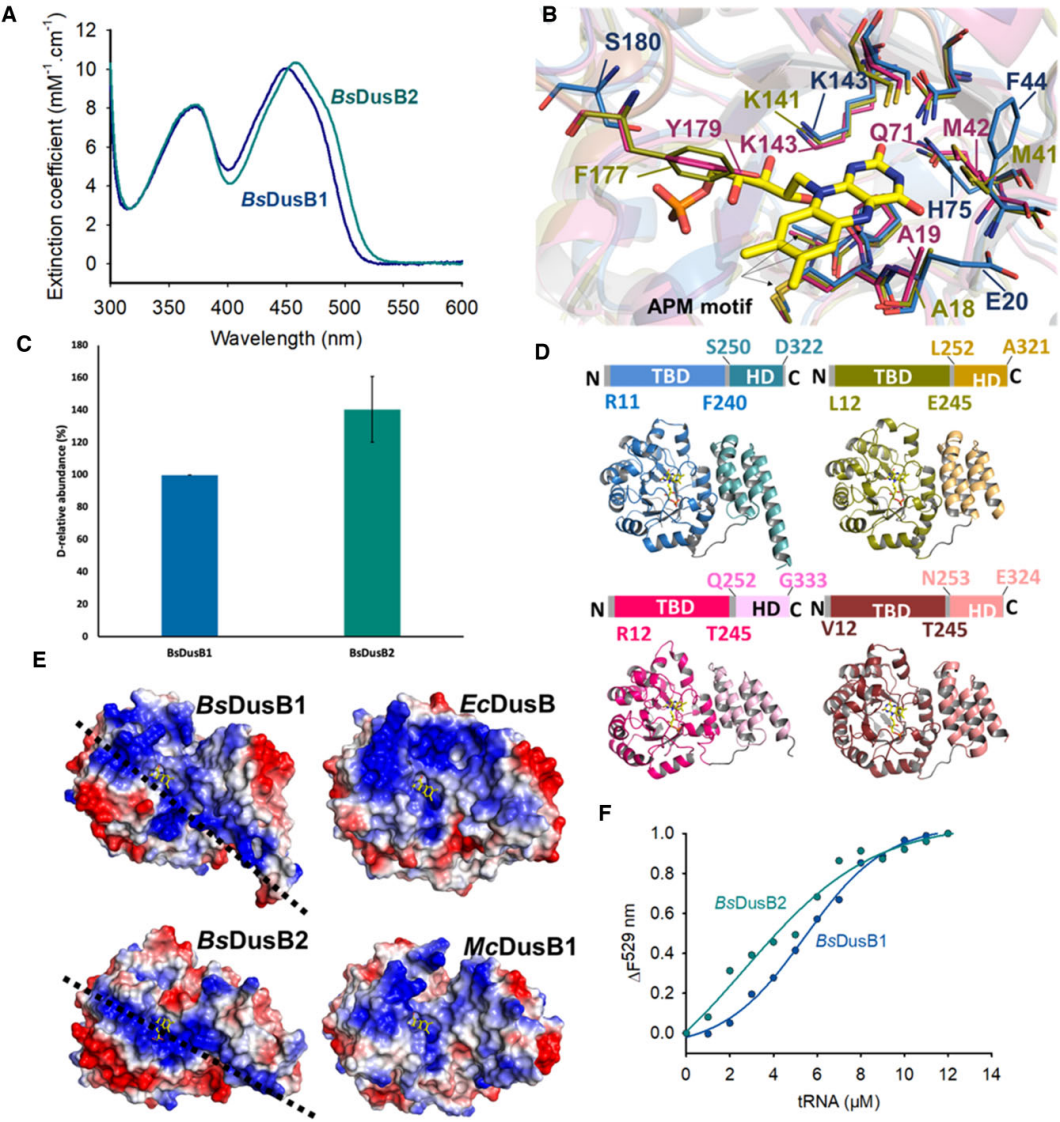
Structural and functional characterization of *B. subtilis* DusB. (**A**) UV-visible absorption spectra of *Bs*DusB1 (blue) and *Bs*DusB2 (teal) holoproteins. (**B**) Comparative structural models of the active sites of *Bs*DusB1, *Bs*DusB2 and DusB of *E. coli* (*Ec*DusB). The active site view is centered on the overlay of a section encompassing the FMN isoalloxazine (yellow) of the respective active site of the three DusB (*Bs*DusB1 in pink, *Bs*DusB2 in blue and *Ec*DusB in deep olive). Residues around the FMN are shown in stick in the respective color codes of the Dus. (**C**) *In vitro* dihydrouridylation activity test of recombinant *Bs*DusB at 1μM of enzyme after 1 hour incubation at 37°C. Dihydrouridine levels were determined by LC-MS/MS and normalized to the UV signal of adenosine. To compare the activity of *Bs*DusB, the activity of *Bs*DusB1 was set to 100%. Results are shown as average of biological duplicates. (**D**) Structural models of the DusB holoenzymes from *B. subtilis*, *E. coli* and *M. capricolum*. Except for *Ec*DusB, which is a crystallographic structure (PDB, 6EI9), the other three models are from Alphafold. TBD = TIM Barrel Domain, HD = Helical domain. The FMN is shown in yellow stick. (**E**) Electrostatic surface of the Dus model. The dashed line represents the line of demarcation (LOD) mentioned in the text. (**F**) Isotherm of tRNA binding to *Bs*DusB. ΔF529nm is the change in FMN fluorescence at 529 nm resulting from tRNA titration to *Bs*DusB1 (blue) and *Bs*DusB2 (teal).

The *Bs*DusB1 and *Bs*DusB2 proteins share a relatively low sequence identity of 26% (Supplementary Figure S3). To characterize these two proteins *in vitro*, the genes encoding *Bs*DusB1 (BSU00810, Uniprot Id P37567) and *Bs*DusB2 (BSU08030, Uniprot Id O31546) were cloned into expression vectors, expressed in *E. coli*, and subsequently purified to homogeneity (Supplementary Figure S4). To determine their oligomeric state in solution, gel filtration on a Superdex increase 75 10/300 column was performed, revealing that both proteins exist as monomers with an estimated molecular weight (Mw) of approximately 40 kDa for *Bs*DusB1 (elution volume ∼ 11.2 ml) and 39 kDa for *Bs*DusB2 (elution volume ∼ 11.7 ml). *Bs*DusB proteins were found to be copurified with their flavin coenzyme, evident from the yellowish color of the protein samples and characteristic absorbance spectra (Figure 5A). The latter featured two absorption bands typical for flavin: the S0-S2 bands exhibited a maximum at 372 nm, while the S0–S1 band in *Bs*DusB1 and *Bs*DusB2 showed a maximum at 450 and 458 nm, respectively. The difference in the wavelength maximum of the S0–S1 transition between the two proteins suggests dissimilarity in the polarity of their active sites. Upon the addition of sodium dodecyl sulfate (SDS), the proteins denatured, releasing flavin into the solution. The resulting flavin in solution displayed an absorption spectrum similar to that of free FMN, confirming that both *Bs*DusB enzymes are flavoproteins with the FMN non-covalently bound to the apoprotein. FMN fluorescence in both holoproteins was also monitored and showed a slight red shift in the maximum fluorescence emission band of *Bs*DusB2, at 530 nm, compared to that of *Bs*DusB1 observed at 527 nm, supporting the existence of distinct environments for the two FMN coenzymes (Supplementary Figure S5). This polarity contrast is substantiated by our analysis of the active sites in the holoprotein forms of *Bs*DusB1 and *Bs*DusB2 Alphafold models (see supplementary results and Figure 5B).

### An unusual behavior of Dus pyrimidine discrimination and dihydrouridylation activity of tRNA

Dus enzymes share a highly conserved catalytic mechanism that involves two redox reactions (22,24). NADPH reduces FMN to yield FMNH^−^, which is then oxidized to upon reduction of uridine to dihydrouridine. We measured the NAD(P)H oxidase activity of the two *Bs*DusB enzymes independently by monitoring the consumption of NADH or NADPH under aerobic conditions using absorbance spectrophotometry at 340 nm and steady-state conditions. The data were analyzed using the Michaelis–Menten formalism and the related kinetics parameters are presented in Table 1. The results revealed that *Bs*DusB1 oxidized NADPH and NADH with identical catalytic constants (*k*_cat_ ∼ 0.013 s^−1^) and comparable *K*_M_ values, indicating that the enzyme did not discriminate between NADH or NADPH and could use both equally. This result was unexpected, because all previously studied Dus enzymes, both prokaryotic and eukaryotic, showed a preference for NADPH over NADH (30,33,43). In contrast, for *Bs*DusB2, NADPH was a better substrate than NADH due to a lower *K*_M_ for NADPH (2 μM) than for NADH (22 μM) and ∼ a 3-fold higher catalytic constant for NADPH than for NADH. Overall, NADPH exhibited a 5-fold higher catalytic efficiency than NADH.

**Table 1.** Kinetic parameters for NAD(P)H oxidase activity of *B. subtilis* Dus

	*NADH*			*NADPH*		
	*k*_cat_ (s^−1^)	*K*_M_ (μM)	*k*_cat_/*K*_M_ (μM^−1^s^−1^)	*k*_cat_ (s^−1^)	*K*_M_ (μM)	*k*_cat_/*K*_M_ (μM^−1^s^−1^)
*Bs*DusB1	0.013 ± 0.0014	21 ± 4	6 × 10^−4^	0.013 ± 0.002	18 ± 3	7 × 10^−4^
*Bs*DusB2	0.23 ±	22 ± 4	6 × 10^−2^	0.7 ± 0.1	2.1 ± 0.2	0.3

To examine the *Bs*DusB activity of *B. subtilis*, *in vitro* dihydrouridylation assays were performed with bulk tRNAs from the double deletion strain, and the reaction products were traced using LC/MS. In the presence of 1 μM protein, *Bs*DusB2 was able to restore a 40% higher D level compared to *Bs*DusB1 after 1 hour, indicating that *Bs*DusB2 is the more active enzyme (Figure 5C).

### Structural characterization of DusB enzymes and RNA binding

The structural models of *Bs*DusB1, *Bs*DusB2, *Ec*DusB and *Mc*DusB1 were examined using models generated through Alphafold Colab2 (Figure 5D). The derived models exhibited per-residue confidence scores exceeding 90% across most of their respective regions, as illustrated in Supplementary Figure S6A. As anticipated, these enzymes display a conserved canonical folding of the Dus family, i.e. (i) a catalytic domain adopting a TIM-Barrel type structure (TBD) where the flavin coenzyme binding site lies at the entrance of the barrel, (ii) a helical domain (HD) composed of a 4-helix bundle, and (iii) a short linker of about 10 amino acids connecting the two domains. Conducting a structural alignment and comparing the models revealed low RMSD values within the *Bs*DusB1 subfamily (Supplementary Figure S6). This supports the notion that the models for *Bs*DusB1, *Mc*DusB1 and *Ec*DusB1 exhibit highly similar structures. A broad distribution of positive surface charges accessible to the solvent, most likely engaged in interactions with the tRNA substrates, can be distinguished (Figure 5E). This distribution is arranged on both sides of a line of demarcation (LOD) that can be drawn from the left extremity of the TBD throughout the active site cavity, ending at the lower tip of the HD at the C-terminus. Several interesting points can be observed based on this spatial arrangement. *Bs*DusB1 has a continuous, positive electrostatic surface stretched on both sides of the LOD, whereas *Bs*DusB2 is distinguished by a positive surface forming an elongated stripe parallel to the LOD and spanning almost on all its length, but primarily found on the proximal side of this line. In *Ec*DusB, a significant portion of the positive area forms an off-center globular area on the distal edge of the LOD, involving predominantly the apical region of the TBD. *Mc*DusB1 shows a certain similarity to *Bs*DusB1 but with a distinctive feature, namely the presence of several rather isolated positive charge patches. Thus, each of the studied DusB seems to have its own tRNA binding pattern, likely adapted to its site specificity. Likewise, each Dus will probably orientate the tRNA in a distinct way to allow the active site of the enzyme to gain access to the correct uridine substrate to be modified (21,44). To evaluate whether this difference in positive surface area affects the stability of the enzyme/tRNA complex, we examined the ability of *Bs*DusB1 and *Bs*Dus2 to bind to tRNA by specifically monitoring the impact of tRNA titration on flavin fluorescence. Addition of tRNA resulted in an increase in FMN fluorescence of both *Bs*DusB describing a cooperative process (Figure 5F). Half-transition was observed at about 3 and 5 μM for *Bs*DusB1 and *Bs*DusB2, respectively, showing no significant differences.

### RNA dihydrouridylation broadening specificity depends on enzyme concentration

Our complementation results in the *E. coli* triple *dus* mutant strain (Δ*dusA*::kan, Δ*dusB*::Ø, Δ*dusC*::Ø) with *BsdusB1* or *BsdusB2* demonstrated that both enzymes could dihydrouridylate positions U17, 20 and 20b, acting as both *Ec*DusB and *Ec*DusA (see supplementary results & Supplementary Figure S7). While the outcomes for *Bs*DusB1 were anticipated, the unexpected capability of *Bs*DusB2 to catalyze D17 formation in *E. coli* was intriguing. This finding suggested several possibilities in a heterologous context: (i) *Bs*DusB2 lost its substrate specificity due to the differences in tRNA nature (sequence and modification profile) between both organisms; (ii) a protein partner, RNA, or other compounds in *B. subtilis* controlled the site specificity; or (iii) the intracellular concentrations of Dus proteins differed between *E. coli* and *B. subtilis*. Indeed, complementation assays in *E. coli* were performed with *BsdusB1* or *BsdusB2* under the control of an arabinose-inducible promoter with concentration of inducer adjusted to allow for the detection of dihydrouridylation. In contrast, in *B. subtilis* both genes are expressed from the chromosome by their own promoter.

**Figure 6.**
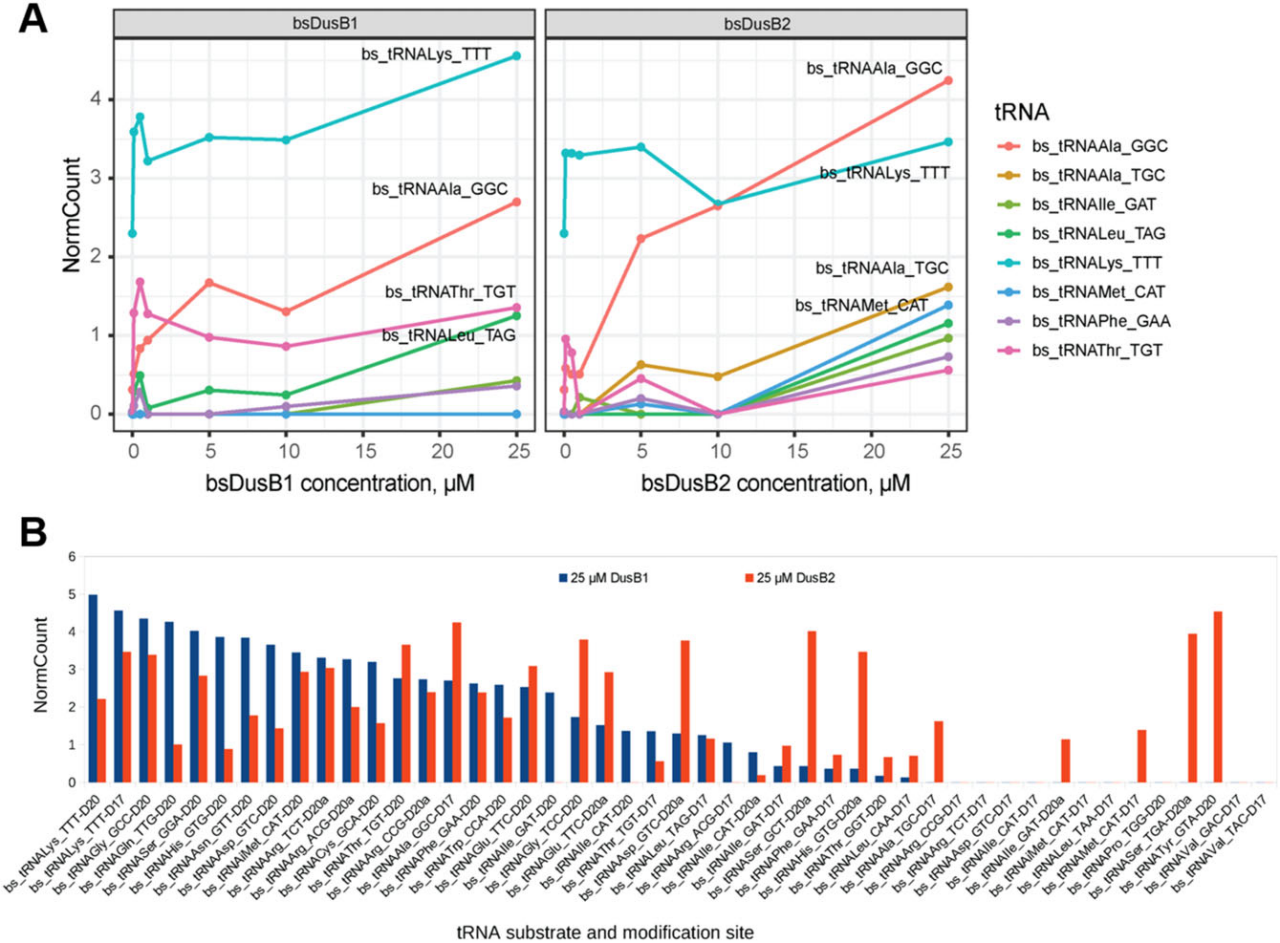
*In vitro* biosynthesis of D in *B. subtilis* tRNAs catalyzed by the recombinant *Bs*DusB1 and *Bs*DusB2 proteins. (**A**) Recombinant enzymes expressed in *E. coli* and purified were incubated with D-unmodified *B. subtilis* total RNA fraction extracted from Δ*dusB1::kan*,Δ*dusB2::erm* strain. Quantification of D17 level was done using NormCount score of AlkAnilineSeq (the signal normalized to median of background cleavages in the surrounding 10 nucleotides). NormCount score (as well as other AlkAnilineSeq Scores) does not show linear dependence from D content, but provides good compromise between sensitivity and specificity of detection for low D levels in tRNA. Only 8 best modified tRNA sites are shown (out of 18 altogether). Concentration of the recombinant *Bs*DusB1 and *Bs*DusB2 is expressed in μM. Identity of tRNA substrates analyzed is shown at the right. (**B**) Modification efficiency of the D-sites measured at 25 μM of enzymes. Quantification of D level was done using NormCount score of AlkAnilineSeq.

To further explore these possibilities, we assessed the effect of increasing enzyme concentrations on *Bs*DusB’s dihydrouridylation activity *in vitro* using tRNA from the *B. subtilis* double deletion strain as a substrate. Additionally, we performed the experiments in the presence of *B. subtilis* Δ*dusB1::kan*, Δ*dusB2::erm* cell extract to examine the existence of a potential partner for *Bs*DusB2 that might be essential for its site specificity. AlkAnilineSeq quantifications showed that the level of D17 inserted by *Bs*DusB1 and *Bs*DusB2 increased with enzyme concentration (Figure 6A), confirming that *Bs*DusB2 can synthetize D17 *in vitro* on tRNA from *B. subtilis*. AlkAnilineSeq also provided insights into the dihydrouridylation efficiency for all D-sites (Figure 6B). Dihydrouridylation efficiency seemed to depend on the nature of the tRNA and the modified position. As expected, *Bs*DusB1 formed D17/D20/D20a. Except for$\ {\mathrm{tRNA}}_{{\mathrm{UUU}}}^{{\mathrm{Lys}}}$ and ${\mathrm{tRNA}}_{{\mathrm{GGC}}}^{{\mathrm{Ala}}}$, the dihydrouridylation efficiency was higher at positions 20 and 20a than at position 17. Experiments conducted with crude *B. subtilis* extracts revealed that *Bs*DusB2 retained its ability to synthesize D17 even at higher enzyme concentrations (data not shown), suggesting the absence of a cellular partner that regulates the specificity of this Dus enzyme. To validate these findings *in vivo*, both wild type and mutant strains were transformed with plasmids overexpressing either *Bs*DusB1 or *Bs*DusB2. AlkAnilineSeq profiles from these strains clearly demonstrated that overexpression of *Bs*DusB2 in dusB1-deficient strains or *Bs*DusB1 in dusB2-deficient strains was able to restore the dihydrouridylation profile for a significant subset of tRNAs (Supplementary Figure S8). Moreover, *Bs*DusB2 exhibited the capability to introduce D17 residues into several tRNAs, indicating its functional equivalence to *Bs*DusB1 upon overexpression (Supplementary Figure S8). Taken together, these results demonstrate that specificity likely depends on both the nature of the tRNAs and the enzyme concentration.

### Effect of *Bs*DusB deletions on cell growth

The optimal growth temperature of *B. subtilis* ranges from 35 to 37°C. The influence of the lack of *Bs*DusB and by extrapolation of D on the growth of *B. subtilis* was investigated in LB medium at 23, 30 and 37°C (Table 2). At the standard growth temperature of 37°C, *B. subtilis* W168 exhibited a generation time of 21 minutes. However, in the case of the three strains with deletions in either one or both *dus* genes, there was a slight increase in generation time. The effect was slightly more visible when cells were grown at 30°C, with the generation time rising from 31 min for the wild type to 39 and 40 min for *ΔdusB1* and *ΔdusB2*, respectively. The effect was even more pronounced in the double mutant strain, where this doubling-time increased to 43 min. A more significant difference in growth was observed when the temperature was lowered to 23°C. Here, the generation time increased from 49 minutes for W168 to 87 min for the three mutant strains. Thus, the absence of D does not seem to have too great of an impact on *B. subtilis* at physiological growth temperatures, but becomes significant at low temperature such as 23°C. This observation aligns with the role of this modified base in promoting structural flexibility at the tRNA level, a feature that is more crucial at lower temperatures than at higher ones.

**Table 2.** Effect of *dus* deletion on the generation time of *B. subtilis*

	37°C	30°C	23°C
W168	21 ± 0.2	31 ± 0.8	49 ± 3
ΔdusB1ΔdusB2	25 ± 0.3	39 ± 2.2	87 ± 0.7
ΔdusB1	26 ± 0.7	40 ± 4	87 ± 2
ΔdusB2	26 ± 0.6	43 ± 1.5	87 ± 1.5

*The generation times are expressed in minutes.

Generation time is just one among several growth parameters for bacteria, serving as an indicator of potential fitness loss. Therefore, we conducted competition experiments between mutants and the wild-type strain to evaluate the impact of tRNA dihydrouridylation loss on mutant fitness. Surprisingly, all mutant strains exhibited decreased fitness compared to the wild type, even at 37°C, with the *ΔdusB1* strain showing the lowest competitive index (Supplementary Figure S9). However, observed differences in fitness among mutants were not statistically significant (*t*-test, *P* > 0.03), suggesting a potential role of the redundancy in specificity of *Bs*DusB enzymes.

## Discussion

We investigated the role of the two homologs, DusB1 and DusB2, in D base biosynthesis in *B. subtilis* tRNAs. Both *Bs*DusB enzymes are FMN-dependent flavoenzymes with a conserved canonical structure of bacterial Dus, retaining key catalytic residues (Figure 5A,B). However, they differ in the polarity of their active sites and preference for the reducing agent, NAD(P)H (see supplementary discussion and Table 1). Most modification enzymes are highly site-specific and modify only one position. However, a small number of enzymes exhibits promiscuous site specificity, targeting either adjacent bases, or multiple positions scattered along the nucleotide sequence of their RNA substrate, or even have both capabilities (45–52) (see also Supplementary Table S4). The Dus enzymes also display the two cases of targeting juxtaposed uridines as observed with bacterial DusA (31) and *Bs*DusB2 for U20–U20a, and with eukaryotic Dus1 (U16–U17) and Dus4 (U20a–U20b)(20). Gram^+^ Dus enzymes show a wider multisite specificity as seen with the *Mc*DusB1 that modifies the U17–U20–U20a triplet (33) and reinforced here with the discovery that *Bs*DusB1 modifies not only the same bases as *Mc*DusB1 but also the U47 (Figure 3). D47 is located in the variable loop which, in eukaryotes, is catalyzed by Dus3, an enzyme that differs from all Dus by its size and complex modularity (22).

Remarkably, we uncovered an unprecedented property in modification enzymes namely, functional redundancy. This property remains very enigmatic since *Bs*DusB1 can introduce almost the entire D content while *Bs*DusB2 provides a backup activity for positions 20–20a with an efficiency largely in favor of this enzyme. It is worth mentioning that this overlap in activity concerns most tRNAs (Figure 4). Nevertheless, *Bs*DusB2 also have its proper tRNA substrates not shared by *Bs*DusB1 suggesting that this D20–D20a redundancy in dihydrouridylation activity targets a specific set of tRNAs. Surprisingly, *Bs*DusB2 has also the ability to modify U17 only at a certain enzyme concentration, which could probably be consistent with a lower dihydrouridylation efficiency for this site (Figure 6). Of note, Dus enzymes involved exclusively in D20 (or D20–D20a) modification seem to be always more active than those catalyzing other D (22) and this has indeed remained verified again with *B. subtilis* enzymes. A recent analysis of dihydrouridylation in the *S. pombe* transcriptome found instances where the dependence of several tRNA sites on Dus enzymes couldn’t be statistically determined (23). This suggests either the necessity of a D-site at a position for modifying another site or rare cases where multiple Dus enzymes target the same site. This could imply broader dihydrouridylation redundancy among Dus enzymes, requiring further clarification.

The physiological significance of this redundancy in *B. subtilis* raises intriguing questions. In general, homolog-based functional redundancy can provide functional resilience or flexibility to cope with varying conditions or stresses (53–55). This could indeed apply to *Bs*DusB taking the advantage of having one enzyme more efficient than the other, especially when dealing with redox reactivity issues. It is tempting to propose that this backup functionality could be a more efficient way to dihydrouridylate tRNAs under conditions or events leading to significant tRNA damage requiring rapid maturation of newly transcribed pools of tRNAs to afford the cell to cope with abrupt environmental changes notably under limiting NADPH concentration for example. In such a scenario, up-regulation of *Bs*DusB2 could also be an additional mean by which the cell boosts tRNA-dihydrouridylation activity but also extends its site specificity to compensate for the low *Bs*DusB1 activity. Interestingly, such type of regulation has precedent as exemplified by the downregulation of the gene coding for the mesophilic *Clostridium botulinum* DusB homolog during a heat shock stress at 45°C (56). In that specific case, D has probably no utility at high temperatures, and thus this bacterium would naturally require less D and would therefore decrease the expression of its cognate enzyme. DusC is also differentially regulated in response to the growth temperature in the thermophilic *B. manusensis* (57). In *B. subtilis*, our studies revealed a visible impact of the absence of *Bs*DusB1 or *Bs*DusB2 on the growth phenotype of this organism (Table 2), suggesting that loss of D can have significant effects on cell physiology.

Another speculative yet intriguing possibility for this functional redundancy is related to the evolutionary process of these enzymes. Both *Bs*DusB1 and *Bs*DusB2 originated from a duplication event of an ancestral Dus enzyme likely multi-site specific. *Bs*DusB1 has retained the functional features of this ancestral enzyme, while *Bs*DusB2 might be undergoing a process of functional speciation. This could explain why *Bs*DusB2’s dihydrouridylation activity at position 17 is detectable only under high enzyme concentration (Figure 6). Comparative analysis of the presumed tRNA binding interfaces on *Bs*DusB models suggests that the *Bs*DusB2 interface is clearly different from the others with nonetheless some positive charges that remain common to these enzyme systems (Figure 5D). This agrees with the fact that *Bs*DusB2 may preferentially bind its tRNAs according to its own recognition mode. Previous phylogenetic analyses proposed that DusB/DusB1 was the common ancestor to all bacterial Dus proteins (34), a finding that we reproduce in a small-scale analysis with reference bacterial genomes (Figure 7). While the exact timing of the branching of the DusB2 subgroup from the DusB group remains uncertain, it is clearly distinct from both the DusA and DusC subgroups. Further comprehensive phylogenetic analyses would be needed to understand this evolutionary relationship. However, this data suggests that *Bs*DusB2 might be converging towards DusA-type activities, specifically modifying the 20/20a position, while potentially losing its capacity to modify the U17 positions like its DusB homologues. This suggests a possible transitional state in the evolution of *Bs*DusB2’s enzymatic specificity.

## Supplementary Material

gkae325_Supplemental_Files

## Data Availability

All NGS data associated to this manuscript are deposited and made publicly available in ENA (The European Bioinformatics Institute EMBL-EBI) under the accession number PRJEB74134. Phylogenetic analysis of DusB1, DusB2, DusC and DusA proteins in 120 reference and complete Bacteria. DusA proteins are in blue. DusC proteins are in green. DusB/DusB1 proteins are in black. DusB2 proteins are in red. The BV-BRC annotations seem to correctly group the proteins with one exception, the Caur_0210 protein annotated as DusB but clustering with the DusB2 proteins. This section of the tree has however very low bootstrap values as the thickness of the tree branches are reflective of the bootstrap percentage values. *E. coli* proteins are highlighted in yellow and *B. subtilis* proteins in purple.
