# Correction to ‘Functional redundancy in tRNA dihydrouridylation’

**DOI:** 10.1093/nar/gkaf185

**Published:** 2025-03-07

This is a correction to: Claudia Sudol *et al.*, Functional redundancy in tRNA dihydrouridylation, *Nucleic Acids Research*, Volume 52, Issue 10, 10 June 2024, Pages 5880–5894, https://doi.org/10.1093/nar/gkae325

In the original publication of this paper, the fourth author was incorrectly listed as Quentin Thullier. The listing has been corrected to read: “Quentin Thuillier”.

