# Supplementary material for: Functional redundancy in tRNA dihydrouridylation
Source: Nucleic Acids Res. 2024 Apr 29;52(10):5880–94. doi: 10.1093/nar/gkae325 (PMC11162810; doi:10.1093/nar/gkae325)
Supplement: gkae325_Supplemental_Files [file gkae325_supplemental_files.zip › Revised Supp Dus Bacillus subtilis For NAR final.docx]

**Functional redundancy in tRNA dihydrouridylation**

Claudia Sudol^1,2^, Lea-Marie Kilz^3^, Virginie Marchand^4,5^, Quentin Thullier^4,5^, Vincent Guérineau^6^, Catherine Goyenvalle^1^, Bruno Faivre^2^, Sabrine Toubdji^1,2^, Murielle Lombard^2^, Olivier Jean-Jean^1^, Valérie de Crécy-Lagard^7,8^, Mark Helm^3^, Yuri Motorin^4,5^, Damien Brégeon*^1^, Djemel Hamdane*^2^

^1^ Sorbonne Université, CNRS, Institut de Biologie Paris Seine, Biology of Aging and Adaptation, Paris, 75252, France

^2^Collège De France, Sorbonne Université, CNRS, Laboratoire de Chimie des Processus Biologiques, ,11 place Marcelin Berthelot, 75231 Paris Cedex 05, France.

^3^ Institut für pharmazeutische und biomedizinische Wissenschaften (IPBW), Johannes Gutenberg-Universität, Mainz 55128, Germany

^4^Université de Lorraine, CNRS, INSERM, UMS2008/US40 IBSLor, EpiRNA-Seq Core Facility, Nancy, F-54000, France.

^5^Université de Lorraine, CNRS, UMR7365 IMoPA, Nancy, F-54000, France.

^6^Institue de Chimie de Substances Naturelles, Centre de Recherche de Gif CNRS, 1 avenue de la Terrasse, 91198 Gif-sur-Yvette, France

^7^Department of Microbiology and Cell Science, University of Florida, Gainesville, Florida, 32611, USA

^8^University of Florida, Genetics Institute, Gainesville, Florida, 32610, USA

To whom correspondence should be addressed:

Djemel Hamdane, Laboratoire de Chimie des Processus Biologiques, CNRS-UMR 8229, Collège de France, 11 place Marcelin Berthelot, 75231 Paris Cedex 05, France, Tel : +33-(0)1-44271645, Email : [djemel.hamdane@college-de-france.fr](mailto:djemel.hamdane@college-de-france.fr)

Damien Brégeon, Sorbonne Université, CNRS, Institut de Biologie Paris Seine, Biology of Aging and Adaptation,, Paris, 75252, France, Email : [damien.bregeon@sorbonne-universite.fr](mailto:damien.bregeon@sorbonne-universite.fr)

**Supplementary methods**

**Growth curve and competition experiments**

Generation time of wild type and mutant cells at different temperature was established by monitoring OD_600_ during cell growth in LB. For competition experiments, overnight culture from single colonies of wild type strain (W168) or mutant strains were serial diluted in water for titration and assessment of initial ratio of competition inoculation. Competition cultures were inoculated with approximately 100 cells of each preculture and incubated with agitation at 37°C or 23°C for 24h. The final ratio of mutant *vs* wild type cells was monitored by plating appropriate dilution on LB-agar and LB-agar containing kanamycin (40 μg.mL-1) and/or erythromycin (5 μg.mL-1). Plates were incubated overnight at 37°C and the number of CFU was determined. Competitive index was calculated by establishing the final wild type cells vs mutant cells ratio and normalized to the initial ratio for each competition.

**Expression and purification of Dus proteins**

Chemically competent *E. coli* BL21DE3 star cells transformed with pET15b-*dusB1* or pET15b-*dusB2* plasmid were grown in LB (Lysogenic Broth) medium supplemented with ampicillin (100 µg.mL^-1^) at 37°C, until the optical density at 600 nm reached 0.6. Protein synthesis was induced by addition of isopropyl-β-D-thiogalactoside (IPTG) to a final concentration of 250 µM. Cells were grown overnight at 16°C, collected by centrifugation (6,000*xg* at 4°C for 15 min) and stored at -80°C until use. Cells were re-suspended in 50 mM sodium phosphate buffer, pH 8, containing 300 mM NaCl, 5 mM dithiothreitol (DTT), 25 mM imidazole, 10% glycerol (v/v), 50 µM free FMN, 1X EDTA-free protease inhibitor cocktail tablet (Roche) and discontinuously sonicated for 15 min in a water ice batch. Cellular extracts were centrifuged for 1 h at 15,000xg, which yielded a soluble fraction of DusB proteins*.* The soluble fraction was loaded on a Ni-NTA column (Qiagen) previously equilibrated with 50 mM sodium phosphate pH 8 containing 300 mM NaCl, 25 mM imidazole, 10% glycerol (v/v), 100 µM free FMN (buffer A). After extensive wash with buffer A, the protein was eluted with buffer A supplemented with 250 mM imidazole. Fractions containing DusB proteins were pooled and concentrated by ultrafiltration. Protein was loaded onto a HiLoad 16/600 Superdex 75 pg equilibrated with 50 mM Tris-HCl pH 8, 250 mM NaCl, 1 mM DTT. Exchange buffer was conducted on PD-10 Desalting Columns containing Sephadex G-25 resin equilibrated in 50 mM HEPES pH 7.5, 150 mM NaCl and 15% glycerol (v/v). Purity of the proteins was assessed by sodium dodecyl sulphate-polyacrylamide gel electrophoresis (SDS-PAGE). Finally, proteins were concentrated to 6.1 mg.mL^-1^ for *Bs*DusB1 and 26.6 mg.mL^-1^ for *Bs*DusB2, then flash frozen in liquid nitrogen and stored at -80°C until use. Protein concentrations were determined by Bradford assay (Biorad) with BSA used as a standard.

**NAD(P)H Oxidase Activity**

The ability of *Bs*DusB1 and *Bs*DusB2 to oxidize NADH and NADPH under steady state conditions was determined in presence of air, as final electron acceptor, in 50 mM HEPES pH 7.5, 150 mM NaCl and 15% glycerol (v/v). Assays were performed using 1.5 µM of *Bs*DusB1 protein or 300 nM of *Bs*DusB2 in the presence of various concentrations of NAD(P)H ranging from 10 to 320 µM. The amount of NAD(P)H oxidized was monitored by following the decrease of absorbance at 343 nm (ε_343_ = 6.21 mM^-1^.cm^-1^). The initial rate versus NAD(P)H concentration was analyzed according to Michaelis-Menten formalism.

**Steady-state Fluorescence**

Fluorescence spectra of wild type *Bs*DusB1 and *Bs*DusB2 were recorded in a 4/10 quartz cell on an Agilent fluorescence spectrophotometer with excitation and emission slit widths of 5 and 10 nm, respectively and a voltage of 600 V. Proteins were excited at 295 nm, and the resulting emission was monitored from 305 to 700 nm. FMN in proteins was excited at 450 and 458 nm for *Bs*DusB1 and *Bs*DusB2, respectively. The resulting fluorescence was monitored from 460 to 700 nm and 468 and 700 nm for *Bs*DusB1 and *Bs*DusB2, respectively. The fluorescence titrations for tRNA binding experiments were done using the same instrument settings. An incubation of 6 min was achieved after each addition of tRNA, which was varied between 1 and 10 μM, on *Bs*DusB1 or *Bs*DusB2 (2 μM) to reach the equilibrium.

**Detection of dihydrouridine by AlkAniline Sequencing**

About 100 ng of gel purified tRNAs were subjected to AlkanilineSeq (1). Briefly, tRNA was subjected to fragmentation by a mild alkaline hydrolysis for 5 min at 96°C. D rings under these conditions are instable and cleaved. Fragments generated were end-repaired by extensive treatment with alkaline phosphatase to remove both pre-existing 5’-P and 3’-P resulting from alkaline hydrolysis. tRNA fragments were then subjected to aniline treatment, resulting in deprotection of a 5’-phosphate at the N+1 nucleotide, which serves as competent 5’-phosphate for selective ligation of sequencing adapters. Libraries were prepared using the NEBNext® Small RNA Library Prep Set for Illumina® using the manufacturer’s recommendations. Libraries were then qualified, quantified, and multiplexed for high-throughput sequencing using a NextSeq2000 with a 50-bp single-end read mode. Initial trimming of adapter sequence was done using Trimmomatic-0.32 (2) with the default parameters. Alignment to the reference tRNA sequence was done by Bowtie2 (ver 2.4.4) in End-to-End mode and with ‘sensitive parameter’ set. Counting of the mapped reads and positions of their 5’-extremities was performed using awk command (3). Coverage for reference sequence was calculated using samtools mpileup command. 5’-end count was directly used for calculation of AAS scores. Stop-ratio (ratio of reads starting at a given position and total number of passing reads) for every position of the reference sequence was calculated using 5’-end count and coverage data. All other steps of analysis were performed in R-Studio 1.0.143 with R version 3.4.4 .

**Supplementary results**

**Analysis of the holoprotein alphafold models**

Despite repeated efforts at crystallization trials, both *Bs*DusB homologs could not be obtained in crystalline form. To further explore the molecular origin of this spectral divergence, we resorted to Alphafold modeling of the apoproteins of the two *Bs*DusB homologs. Alphafold is known for providing highly accurate models, enabling detailed analysis of protein residue side chain conformations. The placement of FMN in the apoprotein models allowed us to generate holoprotein models by structurally aligning them with the crystallographic structure of *E. coli* DusB (*Ec*DusB) (4, [5](#_ENREF_2)) (**Figure 5B**). In this section, we present a comparative analysis specifically focusing on the active sites of both *Bs*DusB and *Ec*DusB, while a broader analysis of the overall fold will be included in a subsequent section of the manuscript. Remarkably, the FMN is situated within a deep crevice in the catalytic domain, specifically the TIM Barrel, and it is stabilized by a network of polar and non-polar interactions along the coenzyme (phosphate + ribityl + isoalloxazine). Notably, no clashes were detected between the apoprotein residues and the modeled FMN, except for residue F44 in the case of *Bs*DusB2. This particular residue interferes with the pyrimidine moiety of the isoalloxazine, requiring the selection of an alternative rotamer to resolve this issue. Upon close inspection, several structural features that could potentially contribute to the different flavin environments in the two *Bs*DusB variants, as well as *Ec*DusB, were identified. In *Bs*DusB1, the hydroxyl group of Y179 is oriented towards the C9 of the benzene ring of FMN, which is situated approximately 3 Å away. Conversely, in *Bs*DusB2, the corresponding residue is S180, positioned about 8 Å away from FMN. In *Ec*DusB, F177 occupies the same position as these two residues. Another relevant feature involves M42 in *Bs*DusB1 (M41 in *Ec*DusB), which is replaced by F44 in *Bs*DusB2. Although both residues are hydrophobic, the presence of a sulfur atom in the methionine side chain can still influence the spectral properties and reactivity of flavins (6,[7](#_ENREF_4)). The second notable difference relates to Q71 in *Bs*DusB1 (Q70 in *Ec*DusB), which is substituted by H75 in *Bs*DusB2. This glutamine residue is generally conserved in Dus enzymes and likely plays a role in stabilizing the anionic form of flavin hydroquinone (FMNH-) formed by the reduction of oxidized FMN by NAD(P)H ([8](#_ENREF_5)). The *re*-face of isoalloxazine is stabilized by the consensus -APM-, A16P17M18, A17P18M19 and A15P16M17 for *Bs*DusB1, *Bs*DusB2 and *Ec*DusB, respectively. However, it is intriguing to observe that the residue following this consensus sequence differs between the two *Bs*DusB, with A19 and E20 for *Bs*DusB1 and *Bs*DusB2, respectively. These residues are facing the N5 nitrogen of isoalloxazine, a key atom in the flavin reactivity.

***Bs*DusB complements *Ec*DusA and *Ec*DusB but not *Ec*DusC**

Although structural analysis of the DusB models revealed distinct patterns in the shape of the tRNA interaction interface, except for DusB1 from *B. subtilis* and *M. capricolum*, which show some similarities, it cannot be ruled out that the nature of the tRNAs in terms of sequences as well as structures could also contribute to the enzymatic site specificity. In this context, we used complementation experiments of the triple mutant *E. coli* strain (∆*dusA::kan,* ∆*dusB::**Ø,* ∆*dusC::Ø*) with recombinant vectors pBAD24::*BsdusB1* or pBAD24::*BsdusB2* followed by MALDI-MS analysis of purified tRNA fragments digested by RNAseA or RNAseT1. Three tRNAs, namely $\text{tRNA}_{\text{CAG}}^{\text{Leu1}}$ for D16-D17-D20, $\text{tRNA}_{\text{GAU}}^{\text{Ile1}}$ and $\text{tRNA}_{\text{ICG}}^{\text{Arg2}}$ for D17-D20-20a, were chosen for their ability to cover all dihydrouridylation sites in *E. coli* (**Figure S7**). The analysis of the results led to some very surprising findings. Indeed, both *Bs*DusB (B1 and B2) were able to produce D17, D20 and D20a with efficiencies that seemed to be tRNA dependent. For example, *Bs*DusB1 and *Bs*DusB2 formed D17 in $\text{tRNA}_{\text{ICG}}^{\text{Arg2}}$and $\text{tRNA}_{\text{CAG}}^{\text{Leu1}}$, however *Bs*DusB2 appeared more efficient. Conversely, in the case of $\text{tRNA}_{\text{GAU}}^{\text{Ile1}}$, it was *Bs*DusB1 that looked more efficient at catalyzing the reduction of U17 even though *Bs*DusB2 can also do so. Regarding D20-D20a, *Bs*DusB2 was able to complement these positions perfectly while *Bs*DusB1 did so only partially. In summary, both *Bs*DusB enzymes effectively complemented the activity of *Ec*DusA and *Ec*DusB in *E. coli*, but they did not complement *Ec*DusC.

**Supplementary discussion**

Previous studies on other Dus enzymes (Dus2p in *S. cerevisiae*, hDus2 in humans, and *Mc*DusB1) revealed a strong preference for NADPH ([9-1](#_ENREF_6)1), indicating the importance of the 2’-phosphate group at the 2’OH ribose of the dinucleotide in the discrimination mechanism. Surprisingly, our findings show that *Bs*DusB1 is the first Dus enzyme with no preference between NADPH and NADH, displaying similar catalytic constants and K_M_ (**Table 1**). In contrast, *Bs*DusB2 behaves like other Dus enzymes, exhibiting a marked preference for NADPH. Notably, *Bs*DusB2 shows significantly higher dihydrouridylation activity than *Bs*DusB1. The observed difference in dihydrouridylation efficiency between the two *Bs*DusB enzymes might be attributed to the first step of the mechanism, as evidenced by the difference in NADPH oxidation activity, which is three orders of magnitude greater for *Bs*DusB2 (**Table 1**). In the reductive phase, the nicotinamide must approach the FMN's N5-atom for flavin reduction, while in the oxidative phase, the reduced flavin transfers its hydride to the C6-uridine target within the tRNA via the same N5-atom. This is evident from crystallographic structures of Thermus thermophilus DusA and *Ec*DusC complexed with tRNA *in vitro* transcripts, where the target uridines align against the flavin coenzyme, positioning the C6-uracil carbon to receive the hydride from this active nitrogen ([1](#_ENREF_9)2,[1](#_ENREF_10)3). Our DusB models also indicate that accommodating both NADPH nicotinamide and tRNA uridine simultaneously near the FMN is unlikely (**Figure 5B**). Thus, these enzymes likely utilize a ping-pong mechanism: NADPH reduces the flavin, followed by dissociation of NADP+ to create space for the tRNA substrate.

**Table S1. Strains used in this study**

| **Organism** | **Name** | **Gene Descriptor** | **References** |
| --- | --- | --- | --- |
| *B .subtilis* ^a^ | 168 SB168 | *trpC2* | ([1](#_ENREF_11)4), ([1](#_ENREF_12)5) |
|  | W168 | *rpoB18* | Chastanet's lab |
|  | BKK00810 | *trpC2* *ΔdusB1::kan* | (16) |
|  | BKE00810 | *trpC2* *ΔdusB1::erm* | ([16](#_ENREF_13)) |
|  | BKE08030 | *trpC2* *ΔdusB2::kan* | ([16](#_ENREF_13)) |
|  | BKE08030 | *trpC2* *ΔdusB2::erm* | ([16](#_ENREF_13)) |
|  |  | *trpC2* *ΔdusB1::kan, ΔdusB2::erm* |  |
|  |  | *∆dusB1::SadusB2-kan, ∆dusB2::erm* |  |
|  |  | *∆dusB1::McdusB1-kan, ∆dusB2::erm* |  |
| *E. coli* ^b^ | BW25113 | *(F−, Δ(araD-araB)567, ΔlacZ4787::rrnB-3, λ−, rph-1, Δ(rhaD-rhaB)568, hsdR514)* | ([17](#_ENREF_14)), ([18](#_ENREF_15)) |
|  | BW25113 | *pBAD24:∅* |  |
|  | BW25113 | *ΔdusA::∅,ΔdusB::∅,ΔdusC::kan,* *pBAD24:∅* |  |
|  | BL21(DE3) | *B F– ompT gal dcm lon hsdSB(rB–mB–) λ(DE3 [lacI lacUV5-T7p07 ind1 sam7 nin5]) [malB+]K-12(λS)* | ([19](#_ENREF_16)) |
|  | BL21(DE3) | *pET15b::BsdusB1* |  |
|  | BL21(DE3) | *pET15b::BsdusB2* |  |
|  | BW25113 | *ΔdusA::∅,ΔdusB::∅,ΔdusC::kan, pBAD24::BsdusB1* |  |
|  | BW25113 | *ΔdusA::∅,ΔdusB::∅,ΔdusC::kan, pBAD24::BsdusB2* |  |

| ^a^ | BGSC, Bacillus Genetic Stock Center |
| --- | --- |
| ^b^ | [Coli Genetics Stock Center](http://cgsc.biology.yale.edu/Strain.php?ID=64667) |

**Table S2. Primer used in this study**

| **Name** | **Sequence** | **Tamplate** |
| --- | --- | --- |
| B1subUp-Baureus F | TGAAAGGAGGAGAAAAATTGatggaagacgttacagat | pEX- *SadusB2* |
| Baureus-UP1R | ctctcctttctcgcctgcCTATAATTCAATTTTAACG |  |
| B2subUp-Baureus F | AGGATTTGATTTTTGTTATGatggaagacgttacagat |  |
| Baureus-UP1R | ctctcctttctcgcctgcCTATAATTCAATTTTAACG |  |
| B1usbUp-Myco F | TGAAAGGAGGAGAAAAATTGatgaaaattggcaatatcca | pET15b-*McdusB1* |
| Myco-UP1R | ctctcctttctcgcctgcTTATTCTTCGCGATATTCTT |  |
| B2subUp-Myco F | AGGATTTGATTTTTGTTATGatgaaaattggcaatatcca |  |
| Myco-UP1R | ctctcctttctcgcctgcTTATTCTTCGCGATATTCTT |  |
| DuB1sub 5PL | AGACAGACGACCTTGAGCAAA | gDNA dusB1sub Δ*dusB2::kan* |
| B1subUp-Baureus R | atctgtaacgtcttccatCAATTTTTCTCCTCCTTTCA |  |
| Baureus-UP1F | CGTTAAAATTGAATTATAGgcaggcgagaaaggagag |  |
| DusB2sub 3PR | TTCGATACTCAAAGCAGCATCAG |  |
| DuBsub 5PL | AGACAGACGACCTTGAGCAAA |  |
| B1subUp-Myco R | tggatattgccaattttcatCAATTTTTCTCCTCCTTTCA |  |
| Myco-UP1F | AAGAATATCGCGAAGAATAAgcaggcgagaaaggagag |  |
| DusB2sub 3PR | TTCGATACTCAAAGCAGCATCAG |  |
| DuB2sub 5PL | GTTATATCCTTGCGACAATCATGC | gDNA dusB2sub Δ*dusB1::kan* |
| B2subUp-Baureus R | atctgtaacgtcttccatCATAACAAAAATCAAATCCT |  |
| Baureus-UP1F | CGTTAAAATTGAATTATAGgcaggcgagaaaggagag |  |
| DusB1sub 3PR | GGGTTATGGCGTGTAGAGACA |  |
| DuB2sub 5PL | GTTATATCCTTGCGACAATCATGC |  |
| B2usbUp-Myco R | tggatattgccaattttcatCATAACAAAAATCAAATCCT |  |
| Myco-UP1F | AAGAATATCGCGAAGAATAAgcaggcgagaaaggagag |  |
| DusB1sub 3PR | GGGTTATGGCGTGTAGAGACA |  |
| NestedDusB1subUp | GAA CGT ATT GCT GGC ACT GT | trpC2 Δ*dusB2::erm* |
| NestedDusB1subDn | AAT GTC TAA CGC GTT GTG GT |  |
| NestedDusB2subUp | GGC ATT TTT AGT ATC TCA GC |  |
| NestedDusB2subDn | GCA CGT TAC TTT AGG ATG CA |  |
| DusB1sub in pET15b up | GGC AGC CAT ATG TTC AAA ATC GGA GAT ATT | pEX-*BsdusB* |
| DusB1sub in pET15b dn | GCA GCC GGA TCC TTA TCC TAC TTT TGC ATT |  |
| DusB2sub in pET15b up | GGC AGC CAT ATG ACA GAA AAT TTC TGG CGT |  |
| DusB2sub in pET15b dn | GCA GCC GGA TCC TTA CTC TAT CCC ATC AAG |  |
| DusB1sub in pBAD24 up | aattcaccATGgTCAAAATCGGAGATATTCA | pEX-*BsdusB* |
| DusB1sub in pBAD24 dn | acagccaagcttTTATCCTACTTTTGCATTTTGA |  |
| DusB2sub in pBAD24 up | aattcaccATGgCAGAAAATTTCTGGCGTGA |  |
| DusB2sub in pBAD24 dn | acagccaagcttTTACTCTATCCCATCAAGA |  |
| pDG148_DusB1_fw | ACAATTAAGCTTAAGGAGGAAGCAGGTATGgTCAAAATCGGAGATATTC | pET15b-*BsdusB1* |
| pDG148_DusB1_rev | TAGCTTGCATGCTTATCCTACTTTTGCATTTTG |  |
| pDG148_DusB2_fw | ACAATTAAGCTTAAGGAGGAAGCAGGTATGACAGAAAATTTCTGGCGTG | pET15b-*BsdusB1* |
| pDG148_DusB2_rev | TAGCTTGCATGCTTACTCTATCCCATCAAGATAC |  |
| pDG148_fw_seq | CCTCTGCTAAAATTCCTGAA | pDG148 |
| pDG148_rev_seq | CGATCTTTCAGCCGACTCAA |  |

**Table S3. Distribution of dihydrouridine in *B. subtilis* tRNAs (from Modomics database)**

|  |  |  |  |
| --- | --- | --- | --- |
| Positions | Number of tRNAs | Frequency (%) |  |
| 17 | 10/24 | 41 |  |
| 20 | 13/24 | 54 |  |
| 20a | 4/24 | 16 |  |
| 47 | 1/24 | 4 |  |
|  |  |  |  |
| Number per tRNA | Number of tRNAs | Frequency (%) |  |
| 0D | 2 | 8 |  |
| 1D | 16 | 67 |  |
| 2D | 6 | 25 |  |

**Table S4. Examples of multi-site specific tRNA modifying enzymes**

| Enzymes | tRNA positions | modifications | References |
| --- | --- | --- | --- |
| *Aquifex aeolicus* Trm1 | G23,G27 | m^2,2^G26; m^2,2^G27 | (20) |
| *E. coli* TruA | U38,U39,U40 | Ψ38, Ψ39, Ψ40 | ([21](#_ENREF_18)) |
| Pyrococcus abyssi TrmI | A57,A58 | m^1^A57, m^1^A58 | ([2](#_ENREF_19)2,[23](#_ENREF_20)) |
| S. cerevisiae Trm7p | N32, N34 | N^m^32, N^m^34 | ([24](#_ENREF_21)) |
| Thermoplasma acidophilum ArcTGT | G13, G15 | G^+^13, G^+^15 | ([25](#_ENREF_22)) |
| S. cerevisiae Pus1p | U26,27,28,34,35,36,65,67 | Ψ26,27,28,34,35,36,65,67 | ([26](#_ENREF_23)) |
| S. cerevisiae Trm4 | C34,40,48,49 | m^5^C34,40,48,49 | ([27](#_ENREF_24)) |


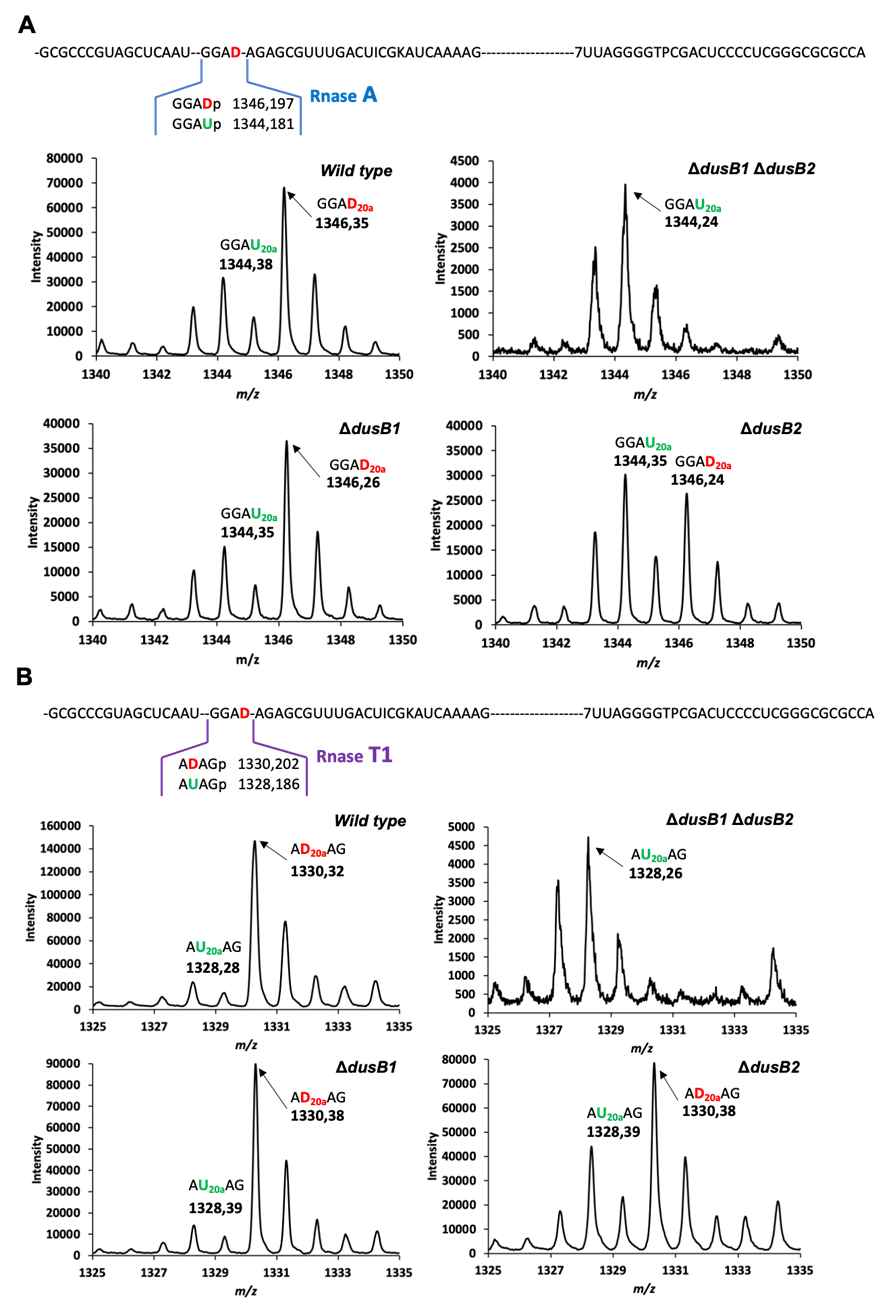


**Figure S1. MALDI-TOF analysis of position 20a in tRNA^Arg^.** (A) D20a-containing MS relative isotope patterns of derived oligonucleotides after RNAse A treatment of $\text{tRNA}_{\text{ICG}}^{\text{Arg}}$ isolated from wild type, Δ*dusB1*Δ*dusB2*, Δ*dusB1 and* Δ*dusB2,* respectively. (B) D20a-containing MS relative isotope patterns of derived oligonucleotides after RNAse T1 treatment of $\text{tRNA}_{\text{ICG}}^{\text{Arg}}$ isolated from wild type, Δ*dusB1*Δ*dusB2*, Δ*dusB1 and* Δ*dusB2,* respectively.


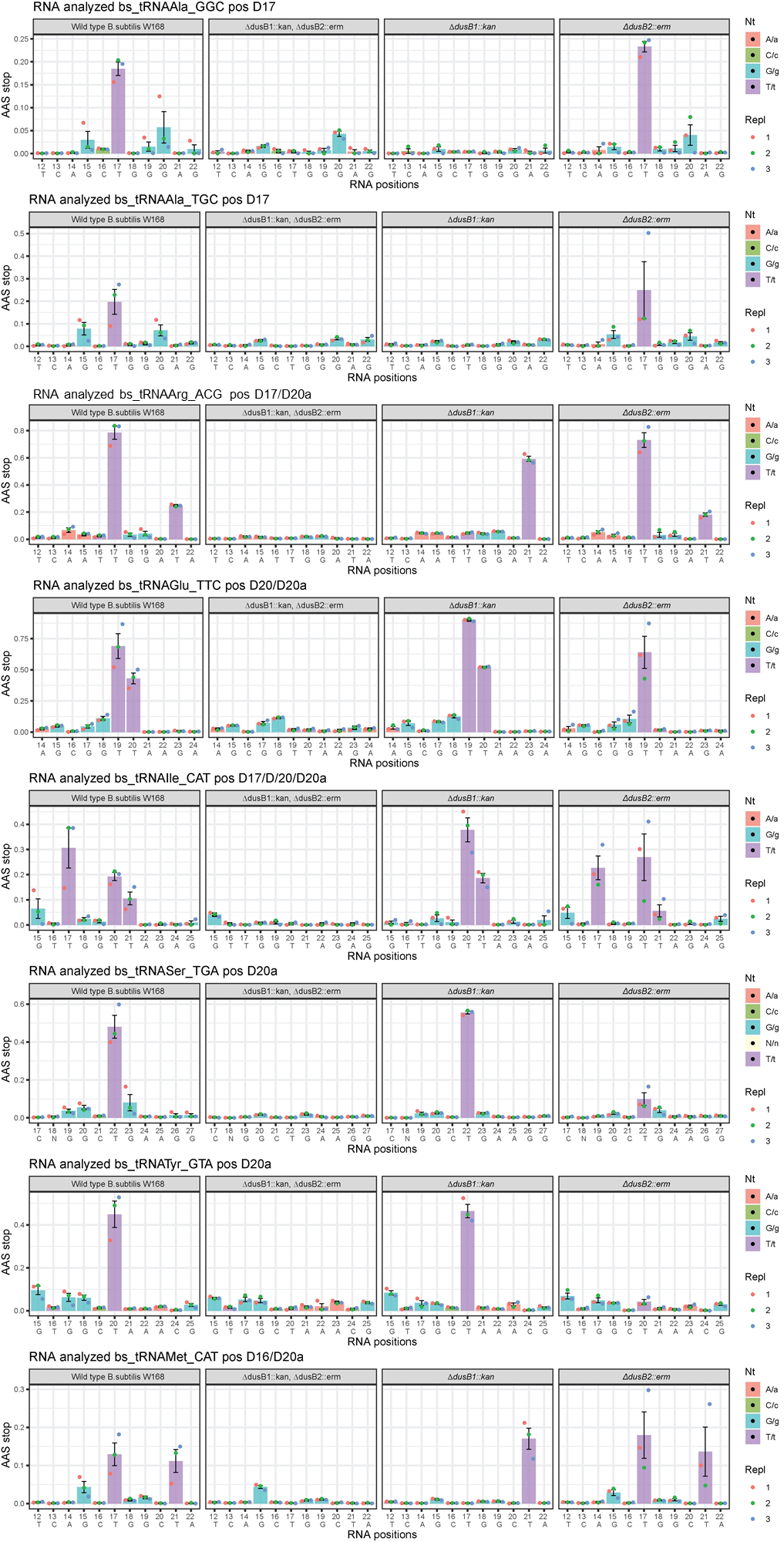


**Figure S2: Analysis of D residues in tRNAs by AlkAnilineSeq method.** AAS stop are shown for selected regions in *B. subtilis* tRNAs of the wild-type strain, the double and single *Bs*DusB mutants. Conventional numbering of tRNA positions may not correspond to real position of nucleotide in the sequence due to missing residues and inclusion of 17a, 20a, 20b, and variable loop nucleotides. Identity of tRNA, its anticodon and the D residues found are indicated on the top of each panel.


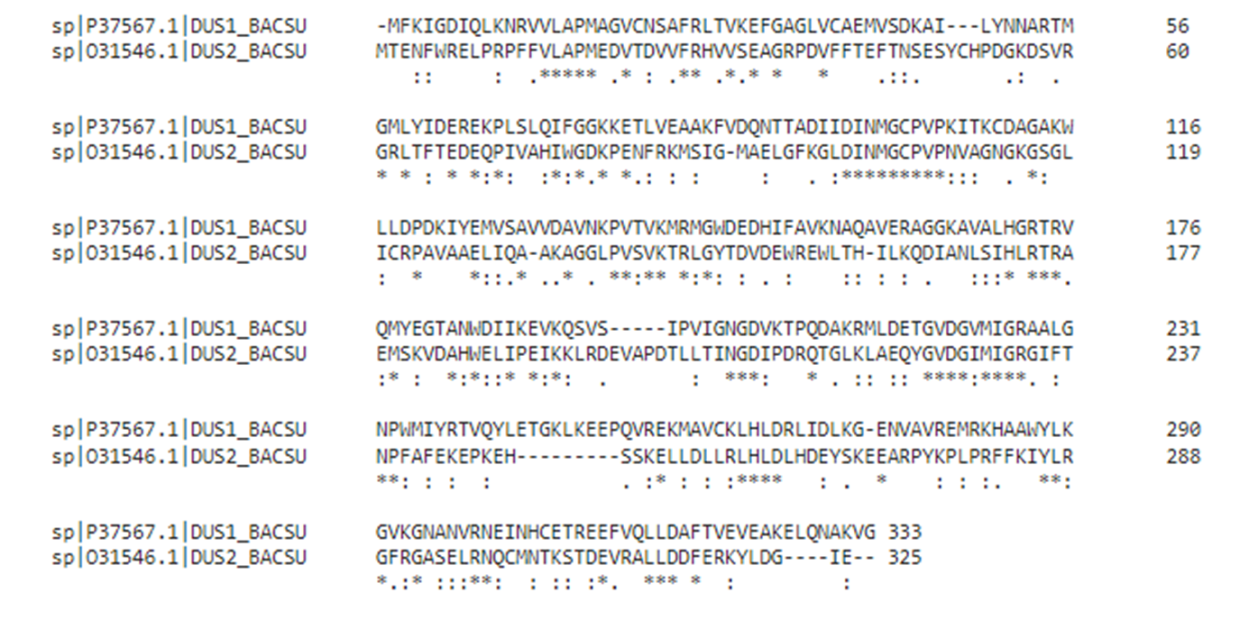


**Figure S3: Sequence alignment of *Bs*DusB1 and *Bs*DusB2 using clustal omega** (https://www.ebi.ac.uk/Tools/msa/clustalo/)


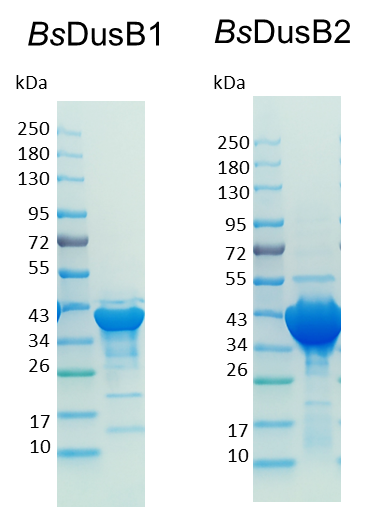


**Figure S4: SDS-PAGE of the recombinant *Bs*DusB after gel filtration**

**Figure S5. Characterization of the active site of *B. subtilis* DusB.** Fluorescence of FMN in holoprotein *Bs*DusB obtained at λex = 450 and 458 nm for *Bs*DusB1 and *Bs*DusB2, respectively.


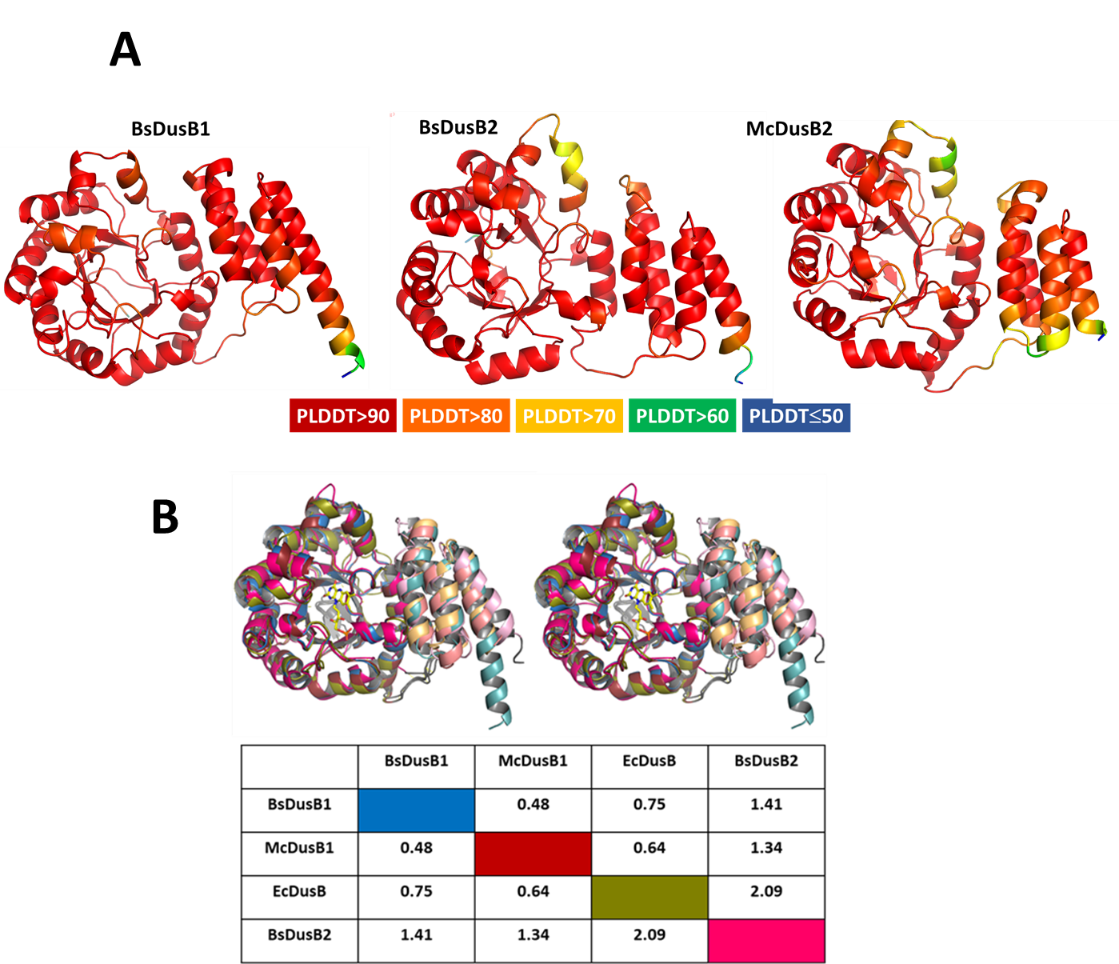


**Figure S6. Structural characterization of DusB enzymes.** (A) AlphaFold models of *Bs*DusB colored according their per-residue model confidence score (pLDDT) between 0 and 100. Regions below 50 pLDDT could be attributed to unstructured structures. (B) Structural alignment of bacterial *Bs*Dus. The models aligned are the DusB holoenzymes from *B. subtilis*, *E. coli* and *M. capricolum*. Except for *Ec*DusB, which is a crystallographic structure (PDB, 6EI9), the other three models are from Alphafold. The table below shows the RMSD in Å for each pair.

**
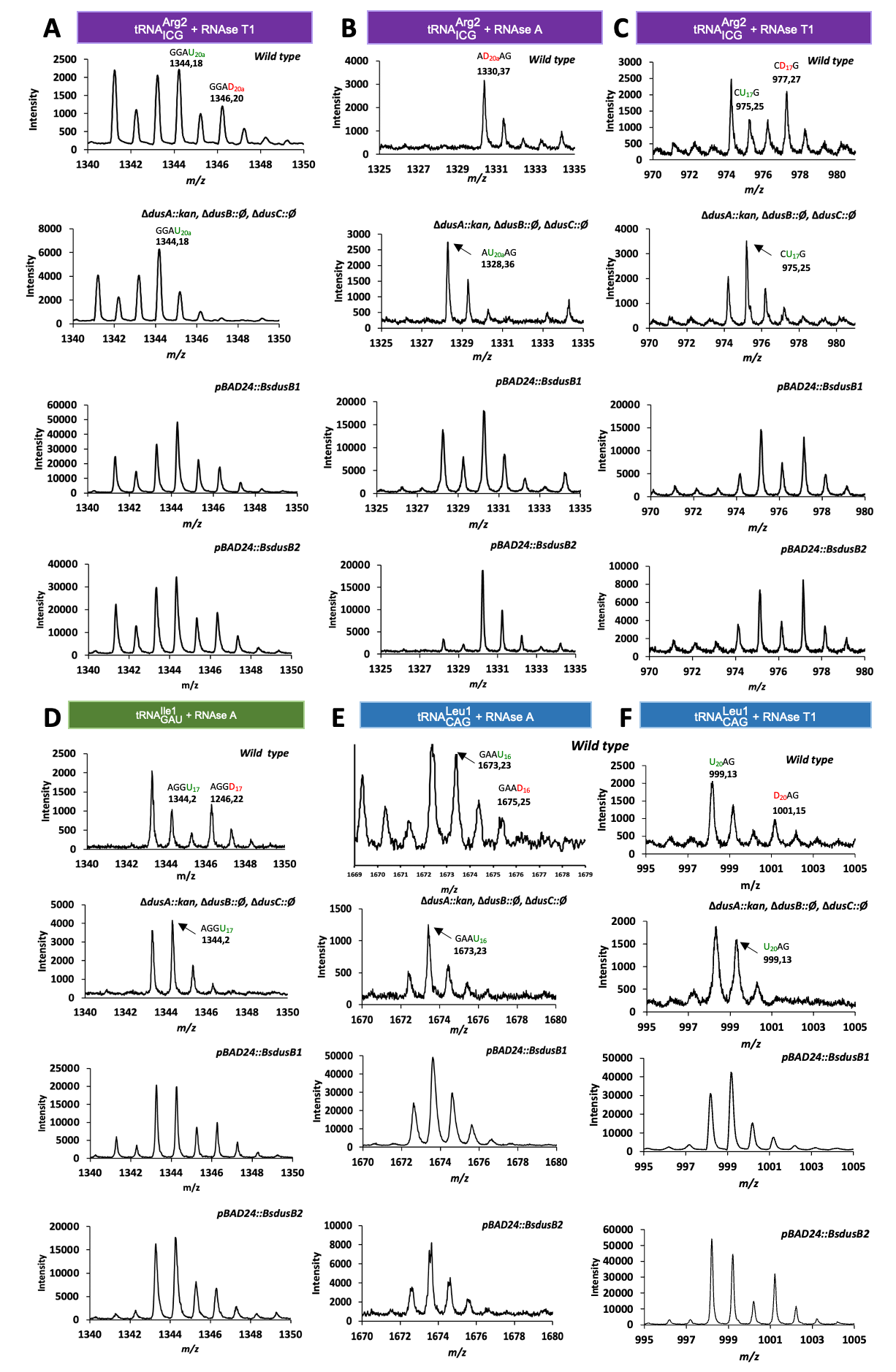
**

**Figure S7. MALDI-TOF analysis of *E. coli* three tRNA extracted from the triple mutant *E. coli* strain (∆*dusA::kan,* ∆*dusB::Ø,* ∆*dusC::Ø*) complement with recombinant vectors pBAD24::*BsdusB1* or pBAD24::*BsdusB2*.** (A-C) MS relative isotope patterns of derived oligonucleotides after RNAseT1 and RNAseA treatment of $\text{tRNA}_{\text{ICG}}^{\text{Arg2}}$ (D) MS relative isotope patterns of derived oligonucleotides after RNAseA treatment of $\text{tRNA}_{\text{GAU}}^{\text{Ile1}}$ (E-F) MS relative isotope patterns of derived oligonucleotides after RNAseT1 and RNAseA treatment of $\text{tRNA}_{\text{CAG}}^{\text{Leu1}}$ The strains are shown in italic while the sequence and m/z of the fragments are in green and red for absence of dihydrouridine or presence, respectively.

**Figure S8. Heatmaps for the assessment of dihydrouridylation changes in individual modified sites in tRNAs from *B. subtilis* and its DusB mutants overexpressing *Bs*DusB1 or *Bs*DusB2.** The heatmap displays one specific D-modification’s stoichiometry across the different samples (in X-axis) and the different D-sites retained for analysis (in Y-axis). The stoichiometry is blue-coded and relies on through stop ratio of the AlkAnilineSeq detection method, which detects m^7^G, m^3^C and D. R1, R2 and R3 represent the results for the three different replicas. Tested strains are indicated above heatmaps. Red boxes show cross complementation of *Bs*DusB2 overexpression in *∆dusB1::erm* strain and black boxes show cross complementation of *Bs*DusB1 overexpression in *∆dusB2::erm* strain.


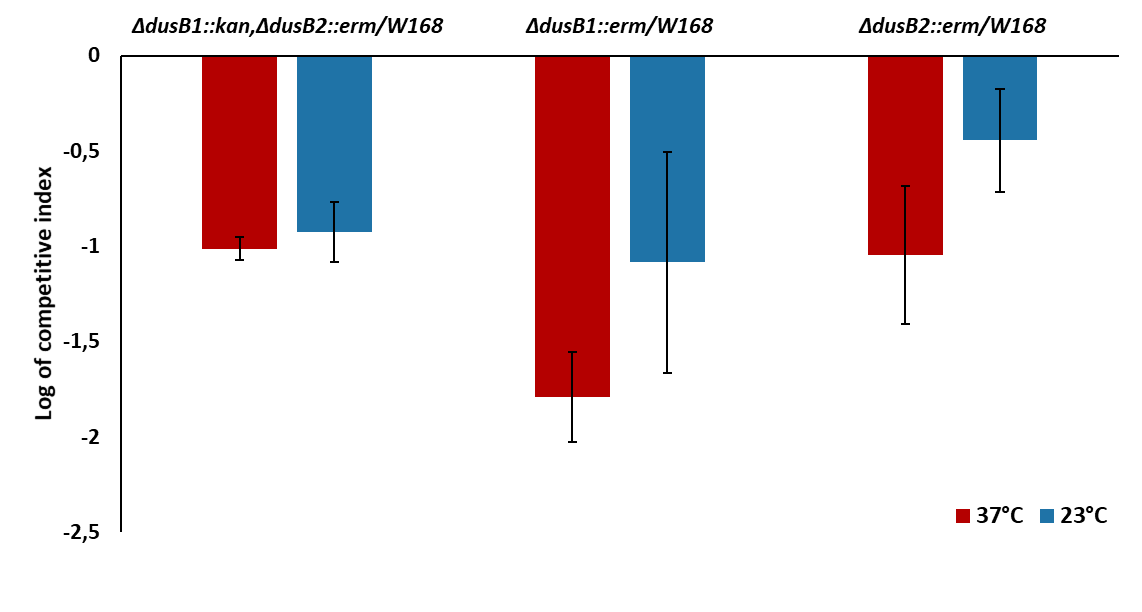


**Figure S9. Growth competition between *B. subtilis* wild type strain (W168) and double or simple mutants at 37°C or 23°C.** Initial number of inoculated bacteria was determined by titration of preculture. After 24 h of incubation, the number of total bacteria was determined by plating on LB-agar plates and the number of mutant cells by plating on LB-agar plates containing erythromycin (∆*dusB1::erm* or ∆*dusB2::erm*) or kanamycin and erythromycin (∆*dusB1::kan*,∆*dusB2::erm*). Competitive index was calculated by dividing the number of mutant cells by the number wild type cells after growth and normalized to the initial ratio. The y-axis represents the Log of competitive index for the indicated competition.

**References**

1. Marchand, V., Ayadi, L., Ernst, F.G.M., Hertler, J., Bourguignon-Igel, V., Galvanin, A., Kotter, A., Helm, M., Lafontaine, D.L.J. and Motorin, Y. (2018) AlkAniline-Seq: Profiling of m(7) G and m(3) C RNA Modifications at Single Nucleotide Resolution. *Angew. Chem. Int. Ed. Engl.*, **57**, 16785-16790.

2. Bolger A.M., Lohse M., Usadel B. (2014) Trimmomatic: a flexible trimmer for Illumina

sequence data. Bioinformatics., **30**, 2114–2120.

3. Marchand V. et al. Next-generation Sequencing-Based RiboMethSeq Protocol for

Analysis of tRNA 2’-O-Methylttion. (2017) Biomolecules **7**, 1-22.

4. Lombard, M., Reed, C.J., Pecqueur, L., Faivre, B., Toubdji, S., Sudol, C., Brégeon, D., De Crecy-Lagard, V. and Hamdane, D. (2022) Evolutionary Diversity of Dus2 Enzymes Reveals Novel Structural and Functional Features among Members of the RNA Dihydrouridine Synthases Family. *Biomolecules*, **12**.

5. Bou-Nader, C., Montemont, H., Guerineau, V., Jean-Jean, O., Brégeon, D. and Hamdane, D. (2018) Unveiling structural and functional divergences of bacterial tRNA dihydrouridine synthases: perspectives on the evolution scenario. *Nucleic Acids Res.*, **46**, 1386-1394.

6. Druhan, L.J. and Swenson, R.P. (1998) Role of methionine 56 in the control of the oxidation-reduction potentials of the Clostridium beijerinckii flavodoxin: Effects of substitutions by aliphatic amino acids and evidence for a role of sulfur-flavin interactions. *Biochemistry*, **37**, 9668-9678.

7. Kasim, M. and Swenson, R.P. (2001) Alanine-scanning of the 50's loop in the Clostridium beijerinckii flavodoxin: Evaluation of additivity and the importance of interactions provided by the main chain in the modulation of the oxidation-reduction potentials. *Biochemistry*, **40**, 13548-13555.

8. Brégeon, D., Pecqueur, L., Toubdji, S., Sudol, C., Lombard, M., Fontecave, M., de Crecy-Lagard, V., Motorin, Y., Helm, M. and Hamdane, D. (2022) Dihydrouridine in the Transcriptome: New Life for This Ancient RNA Chemical Modification. *ACS Chem. Biol*., **17**, 1638-1657.

9. Rider, L.W., Ottosen, M.B., Gattis, S.G. and Palfey, B.A. (2009) Mechanism of dihydrouridine synthase 2 from yeast and the importance of modifications for efficient tRNA reduction. *J. Biol. Chem.*, **284**, 10324-10333.

10. Bou-Nader, C., Pecqueur, L., Brégeon, D., Kamah, A., Guerineau, V., Golinelli-Pimpaneau, B., Guimaraes, B.G., Fontecave, M. and Hamdane, D. (2015) An extended dsRBD is required for post-transcriptional modification in human tRNAs. *Nucleic acids Res.*, **43**, 9446-9456.

11. Faivre, B., Lombard, M., Fakroun, S., Vo, C.D., Goyenvalle, C., Guerineau, V., Pecqueur, L., Fontecave, M., De Crecy-Lagard, V., Brégeon, D. *et al.* (2021) Dihydrouridine synthesis in tRNAs is under reductive evolution in Mollicutes. *RNA Biol.*, **18**, 2278-2289.

12. Yu, F., Tanaka, Y., Yamashita, K., Suzuki, T., Nakamura, A., Hirano, N., Yao, M. and Tanaka, I. (2011) Molecular basis of dihydrouridine formation on tRNA. *Proc. Natl. Acad. Sci. U.S.A.*, **108**, 19593-19598.

13. Byrne, R.T., Jenkins, H.T., Peters, D.T., Whelan, F., Stowell, J., Aziz, N., Kasatsky, P., Rodnina, M.V., Koonin, E.V., Konevega, A.L. *et al.* (2015) Major reorientation of tRNA substrates defines specificity of dihydrouridine synthases. *Proc. Natl. Acad. Sci. U.S.A.*, **112**, 6033-6037.

14. Burkholder, P.R. and Giles, N.H., Jr. (1947) Induced biochemical mutations in *Bacillus subtilis*. *Am. J. Bot.*, **34**, 345-348.

15. Nester, E.W. and Lederberg, J. (1961) Linkage of genetic units of *Bacillus subtilis* in DNA transformation. *Proc. Natl. Acad. Sci. U.S.A.*, **47**, 52-55.

16. Koo, B.M., Kritikos, G., Farelli, J.D., Todor, H., Tong, K., Kimsey, H., Wapinski, I., Galardini, M., Cabal, A., Peters, J.M. *et al.* (2017) Construction and Analysis of Two Genome-Scale Deletion Libraries for *Bacillus subtilis.* *Cell Syst.*, **4**, 291-305.

17. Datsenko, K.A. and Wanner, B.L. (2000) One-step inactivation of chromosomal genes in *Escherichia coli* K-12 using PCR products. *Proc. Natl. Acad. Sci. U.S.A.*, **97**, 6640-6645.

18. Baba, T., Ara, T., Hasegawa, M., Takai, Y., Okumura, Y., Baba, M., Datsenko, K.A., Tomita, M., Wanner, B.L. and Mori, H. (2006) Construction of *Escherichia coli* K-12 in-frame, single-gene knockout mutants: the Keio collection. *Mol. Syst. Biol.*, **2**, 2006.0008.

19. Studier, F.W., Rosenberg, A.H., Dunn, J.J. and Dubendorff, J.W. (1990) Use of T7 RNA polymerase to direct expression of cloned genes. *Methods Enzymol.*, **185**, 60-89.

20. Awai, T., Kimura, S., Tomikawa, C., Ochi, A., Ihsanawati, Bessho, Y., Yokoyama, S., Ohno, S., Nishikawa, K., Yokogawa, T. *et al.* (2009) *Aquifex aeolicus* tRNA (N-2, N-2-Guanine)-dimethyltransferase (Trm1) Catalyzes Transfer of Methyl Groups Not Only to Guanine 26 but Also to Guanine 27 in tRNA. *J. Biol. Chem.*, **284**, 20467-20478.

21. Hur, S. and Stroud, R.M. (2007) How U38, 39, and 40 of many tRNAs become the targets for pseudouridylation by TruA. *Mol. Cell*, **26**, 189-203.

22. Roovers, M., Wouters, J., Bujnicki, J.M., Tricot, C., Stalon, V., Grosjean, H. and Droogmans, L. (2004) A primordial RNA modification enzyme: the case of tRNA (m(1)A) methyltransferase. *Nucleic Acids Res.*, **32**, 465-476.

23. Hamdane, D., Guelorget, A., Guerineau, V. and Golinelli-Pimpaneau, B. (2014) Dynamics of RNA modification by a multi-site-specific tRNA methyltransferase. *Nucleic Acids Res.*, **42**, 11697-11706.

24. Pintard, L., Lecointe, F., Bujnicki, J.M., Bonnerot, C., Grosjean, H. and Lapeyre, B. (2002) Trm7p catalyses the formation of two 2 '-O-methylriboses in yeast tRNA anticodon loop. *EMBO J.*, **21**, 1811-1820.

25. Kawamura, T., Hirata, A., Ohno, S., Nomura, Y., Nagano, T., Nameki, N., Yokogawa, T. and Hori, H. (2016) Multisite-specific archaeosine tRNA-guanine transglycosylase (ArcTGT) from *Thermoplasma acidophilum*, a thermo-acidophilic archaeon. *Nucleic Acids Res.*, **44**, 1894-1908.

26. Motorin, Y., Keith, G., Simon, C., Foiret, D., Simos, G., Hurt, E. and Grosjean, H. (1998) The yeast tRNA:pseudouridine synthase Pus1p displays a multisite substrate specificity. *RNA*, **4**, 856-869.

27. Motorin, Y. and Grosjean, H. (1999) Multisite-specific tRNA:m5C-methyltransferase (Trm4) in yeast *Saccharomyces cerevisiae*: identification of the gene and substrate specificity of the enzyme. *RNA*, **5**, 1105-1118.
